# Tin dioxide-based nanomaterials as anodes for lithium-ion batteries

**DOI:** 10.1039/d0ra10194j

**Published:** 2021-01-04

**Authors:** Minkang Wang, Tianrui Chen, Tianhao Liao, Xinglong Zhang, Bin Zhu, Hui Tang, Changsong Dai

**Affiliations:** School of Materials and Energy, University of Electronic Science and Technology of China Chengdu 611731 China tanghui@uestc.edu.cn; School of Chemistry and Chemical Engineering, Harbin Institute of Technology Harbin 150001 P. R. China changsd@hit.edu.cn

## Abstract

The development of new electrode materials for lithium-ion batteries (LIBs) has attracted significant attention because commercial anode materials in LIBs, like graphite, may not be able to meet the increasing energy demand of new electronic devices. Tin dioxide (SnO_2_) is considered as a promising alternative to graphite due to its high specific capacity. However, the large volume changes of SnO_2_ during the lithiation/delithiation process lead to capacity fading and poor cycling performance. In this review, we have summarized the synthesis of SnO_2_-based nanomaterials with various structures and chemical compositions, and their electrochemical performance as LIB anodes. This review addresses pure SnO_2_ nanomaterials, the composites of SnO_2_ and carbonaceous materials, the composites of SnO_2_ and transition metal oxides, and other hybrid SnO_2_-based materials. By providing a discussion on the synthesis methods and electrochemistry of some representative SnO_2_-based nanomaterials, we aim to demonstrate that electrochemical properties can be significantly improved by modifying chemical composition and morphology. By analyzing and summarizing the recent progress in SnO_2_ anode materials, we hope to show that there is still a long way to go for SnO_2_ to become a commercial LIB electrode and more research has to be focused on how to enhance the cycling stability.

## Abstract 2

The development of new electrode materials for lithium-ion batteries (LIBs) has attracted significant attention because commercial anode materials in LIBs, like graphite, may not be able to meet the increasing energy demand of new electronic devices.

## Graphical abstract

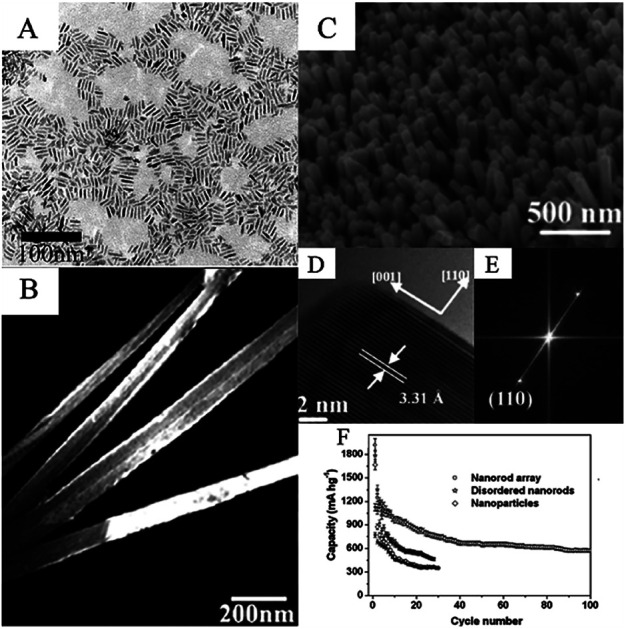

## 1. Introduction

Lithium-ion batteries (LIBs) have been widely used in modern electronic equipment, such as laptops, mobile phones, and mechanical devices for their high energy densities, long cycle life, and environmental friendliness.^1–6^ As an important component of LIBs, electrode materials are responsible for energy storage and cycling life of LIBs.^7–11^ Currently, graphite is the main anode in commercial LIBs owing to its good stability.^12–14^ However, it cannot fully meet the increasing energy demand for batteries due to its low theoretical specific capacity (372 mA h g^−1^).^15–18^ In order to solve this problem, much work has been focused on exploring alternative materials with high specific capacity.^19–26^

Potential candidates like metal oxides have been widely researched due to their high theoretical specific capacities, such as MnO,^27–30^ MnO_2_,^31–36^ Mn_3_O_4_,^37–40^ Fe_2_O_3_,^41–45^ Fe_3_O_4_,^46–50^ Co_3_O_4_,^51–54^ and SnO_2_.^55–57^ Among these materials, SnO_2_ has attracted significant attention due to its low cost, natural abundance, and high theoretical specific capacity (782 mA h g^−1^ of bulk SnO_2_). SnO_2_ is superior to other metal oxides as it has a low charge and discharge potential, *i.e.*, an average charge and discharge potential of 0.5 V and 0.3 V *vs.* Li/Li^+^, respectively,^58^ resulting in LIBs with higher energy density. However, the commercial use of SnO_2_ as an anode material is still hindered by poor cycling stability and inferior rate performance, which is attributed to the electrochemical reaction mechanism of SnO_2_ during lithiation/delithiation. For SnO_2_-based anode material for LIBs, the electrochemistry includes two steps, shown as follows:^59–61^

1 SnO_2_ + 4Li^+^ + 4e^−^ → Sn + 2Li_2_O

2 Sn + *x*Li^+^ + *x*e^−^ ↔ Li_*x*_Sn (0 ≤ *x* ≤ 4.4)

In the first reaction, SnO_2_ reacts with Li^+^ and electrons to generate Sn and Li_2_O. It is believed as an irreversible process, and this is the main reason why SnO_2_ suffers severe capacity deterioration in the initial lithiation process.^62^ In the second reaction, Sn obtained from the first step reacts with Li^+^ and electrons to reversibly generate Li_*x*_Sn alloys. The alloying and dealloying processes represent discharging and charging processes of SnO_2_-based anode material, respectively.^63^ However, Li-alloying anode materials like Li_*x*_Sn and Li_*x*_Si, possess the disadvantages of limited cycle life and severe capacity loss because of large volume changes, pulverization, and continuous formation of solid electrolyte interphase (SEI) during the alloying/dealloying process. Therefore, owing to this irreversible phase transformation process during lithiation/delithiation, the commercial use of SnO_2_ is largely hampered.

In order to solve these problems and to improve the electrochemical performance of SnO_2_, researchers have synthesized many SnO_2_-based anode materials with various well-designed architectures. These SnO_2_-based anode materials can be classified into four types, according to their chemical composition. The first category includes pure nanostructured SnO_2_ materials, such as one-dimensional (1D) nanorods (NDs), nanotubes (NTs)^64–68^ and nanowires (NWs),^69–74^ two-dimensional nanobelts,^75,76^ nanosheets^77–82^ and nanoplates,^83–85^ three-dimensional (3D) hollow nanostructures,^86–92^ and hierarchical nanostructures.^93^ Nanosized materials shorten transmission distance for electrons and Li^+^ and also help to reduce the extent of volume changes during the electrochemical process. In addition, it has been reported that the reduction reaction of SnO_2_ nanomaterials becomes reversible or partly reversible, which improves the lithium storage capacity and reduces the capacity loss during the charge/discharge process.^94^ So the theoretic capacity of SnO_2_ nanomaterials can be increased up to 1495 mA h g^−1^. The second category includes the composites of SnO_2_ and carbonaceous materials,^95–97^ such as SnO_2_/carbon nanotubes (CNTs),^98–101^ SnO_2_/hollow carbon spheres,^102–104^ SnO_2_/graphene^105–110^ and SnO_2_/amorphous carbon. Carbonaceous materials improve the conductivity of the composites and also provide abundant nanosized voids as buffers to decrease the effect of large volume changes during the charge/discharge process.^111–113^ The third category includes the composites of SnO_2_, transition metal oxides, and carbonaceous materials (SnO_2_/TMOs/C). Various composites of SnO_2_ and transition metal oxides (SnO_2_/TMOs) have been synthesized in the past 20 years, such as SnO_2_/Fe_2_O_3_,^114–117^ SnO_2_/Co_3_O_4_,^118,119^ SnO_2_/TiO_2_,^120–122^ SnO_2_/ZnO^123^ and SnO_2_/MoO_3_ (ref. 124) and they showed enhanced lithium storage capacity compared to pure SnO_2_ anode material.^125^ It has been reported that the introduction of TMOs, such as Fe_2_O_3_ (ref. 126) and Co_3_O_4_,^127^ can effectively enhance the capacity because the transition metal nanoparticles in the composite can reversibly convert the extra Li_2_O into Li^+^; thus, influencing the charge/discharge processes. However, cycling stability and rate performance of the composites still need to be further improved. Therefore, based on SnO_2_/TMOs materials, much work has been done to synthesize the composites of SnO_2_, TMOs, and carbonaceous materials (SnO_2_/TMOs/C),^128–131^ like Fe_3_O_4_/SnO_2_/rGO,^132^ SnO_2_@C@Fe_3_O_4_ (ref. 133) and SnO_2_/MoO_3_/C.^134,135^ SnO_2_/TMOs/C materials more effectively alleviate the impact of volume changes and improve conductivity, leading to better electrochemical performance as an anode, compared to the SnO_2_/TMOs materials.^136,137^ The fourth category includes some other tin dioxide-based compounds, such as heteroatom-doped SnO_2_ (ref. 138 and 139) (Fe-doped SnO_2_,^140^ Zn-doped SnO_2_ (ref. 141)), Li_4_Ti_5_O_12_/SnO_2_ (ref. 142) and SnO_2_/C_3_N_4_.^143^

**Scheme 1:**
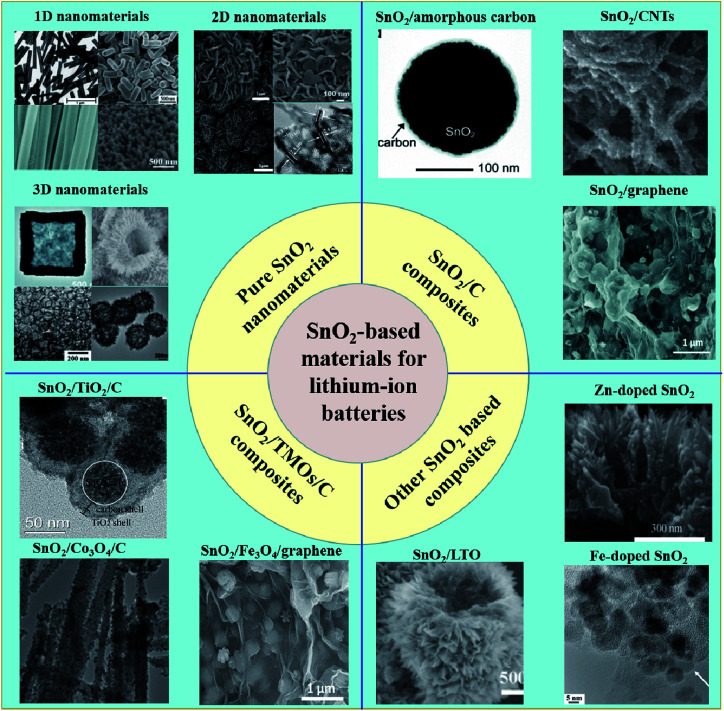
Schematic presentation of different SnO_2_-based anode materials for LIBs.

In this article, we provide the recent progress in the research of these four major types of SnO_2_-based anode material, as mentioned above (Scheme 1). For the following sections, we will introduce the various SnO_2_-based nanomaterials as well as their corresponding synthesis methods and electrochemical performance. We hope this review article will serve as a good reference for further research.

## 2. Pure nanostructured SnO_2_ materials

### 2.1 1D nanomaterials

Many 1D nanostructured SnO_2_ materials, such as nanorods,^144–146^ nanotubes^147–150^ and nanowires^151–153^ have been synthesized in recent years. 1D SnO_2_ nanomaterials usually exhibit superior specific discharge capacity since they can offer extra channels and pathways for electron transmission compared to bulk SnO_2_ and SnO_2_ powders.^154–156^

#### 2.1.1 SnO_2_ nanorods

Pure SnO_2_ nanorods can be synthesized *via* many methods such as chemical vapor deposition (CVD), vapor liquid solid,^157^ hydrothermal treatment^158^ and spray pyrolysis approach.^159^ Among these reported methods, the hydrothermal synthesis of SnO_2_ nanorods is the most used routine as it can be conveniently controlled due to its facile operation. The formation mechanism in a hydrothermal system can be summarized as follows:

3 Sn^4+^ + 4OH^−^ → Sn(OH)_4_ → SnO_2_ + 2H_2_O

First, Sn(OH)_4_ forms by the hydrolysis of Sn-based salts in aqueous medium. During the hydrothermal treatment, Sn(OH)_4_ tends to convert into SnO_2_ and subsequently grows along the [001] direction.^160,161^ Early in 2003, Zhang *et al.* fabricated uniform SnO_2_ nanorods with diameters of about 8–15 nm and lengths of about 150–200 nm by a one-step procedure under mild conditions.^148^ They dissolved sodium dodecyl sulfate and Sn(OH)_6_^2−^ salt in a solution consisting of heptane and hexanol by stirring. Then, the homogeneously dispersed solution was transferred into a Teflon-lined autoclave and heated to 200 °C for 18 h. The as-prepared SnO_2_ nanorods displayed a crystalline rutile structure. Zhang *et al.* also discovered that the concentration of Sn(OH)_6_^2−^ ions and the ratio of NaOH and SnCl_4_ determined the shape of the SnO_2_ nanorods. It was found that on increasing the concentration of Sn(OH)_6_^2−^ from 0.2 M to 0.3 M, the number of nanorods significantly decreased; additionally, on increasing the molar ratio of NaOH to SnCl_4_ from 10 : 1 to 30 : 1, the aspect ratio of SnO_2_ nanorods increased.^162^

**Fig. 1:**
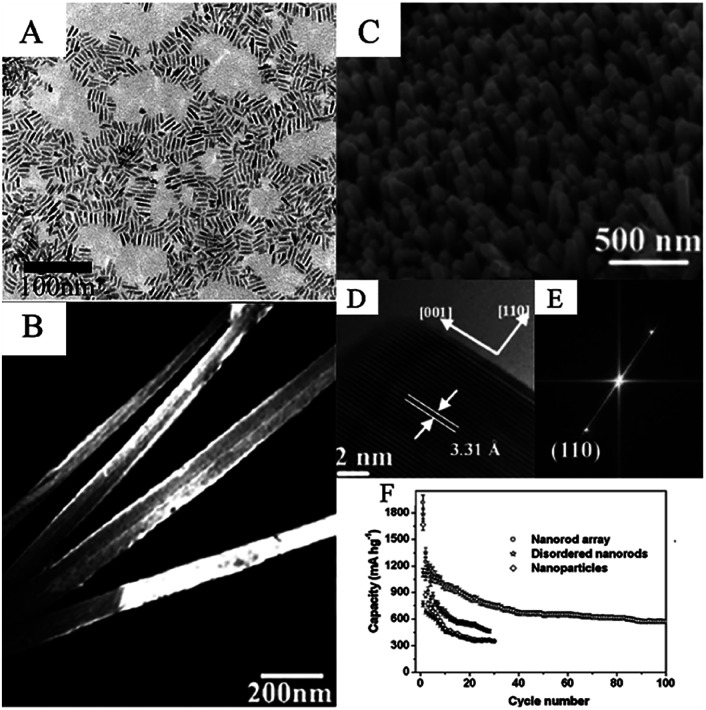
(A) Transmission electron microscopy (TEM) images of the as-prepared SnO_2_ nanorods (adapted with permission from ref. 163 copyright 2004 American Chemical Society). (B) Scanning electron microscopy (SEM) images of long SnO_2_ nanorods (adapted with permission from ref. 58 copyright 2004 Elsevier). (C) SEM images, (D) high-resolution TEM (HRTEM) images, (E) FFT patterns of the HRTEM images, and (F) cycling performance of SnO_2_ nanorods (adapted with permission from ref. 154 copyright 2009 Royal Society of Chemistry).

In 2004, Cheng *et al.* investigated a large-scale hydrothermal method to synthesize single-crystalline SnO_2_ nanorods with lengths of 15–20 nm and diameters of 2.5–5 nm (Fig. 1A). Sn^4+^ precursor was dissolved in a mixture of water and alcohol, and pH was adjusted 12 and the solution was then heated at 150 °C for 24 h.^163^ Based on Zhang and Cheng's work, many groups have synthesized SnO_2_ nanorods *via* hydrothermal methods in other different systems. Guo *et al.* synthesized SnO_2_ nanorods with diameter in the range of 120–260 nm and length up to 2–3 μm by using hexadecyltrimethylammonium bromide as a template (Fig. 1B).^58^ Chen *et al.* synthesized single crystalline SnO_2_ nanorods with diameters of 4–15 nm and lengths of 100–200 nm.^145^ Xi *et al.* investigated a new synthesis method of ultrathin SnO_2_ nanorods with an average diameter of 2 ± 0.5 nm.^164^ Therefore, various SnO_2_ nanorods with distinct morphology can be synthesized by controlling hydrothermal conditions.

SnO_2_ nanorods can be applied as anode materials in LIBs.^165^ Liu *et al.* synthesized SnO_2_ nanorods arrays on a flexible alloy substrate *via* hydrothermal method. The nanorods arrays have average diameter and length of 60 and 670 nm, respectively (Fig. 1C). Observed by HRTEM images (Fig. 1D) and FFT pattern of HRTEM images (Fig. 1E), SnO_2_ nanorod array was growing on substrate along [001] direction, since (001) plane is more loosely packed and has a relatively high surface energy compared to {110} planes.^166^ As-collected hierarchical array structure can directly be used as a binder-free electrode for LIBs, which shows good energy performance, including high discharge capacity (the first discharge is 1918 mA h g^−1^) and good cycling stability (580 mA h g^−1^ after 100 cycles at 0.1C, with coulombic efficiency of nearly 100%) (Fig. 1F).^154^

#### 2.1.2 SnO_2_ nanotubes

SnO_2_ nanotubes have been widely used as anode materials in lithium-ion batteries. Compared to SnO_2_ nanorods, SnO_2_ nanotubes usually possess superior lithium storage capacity and cycling stability due to their hollow characteristics.^167^ Sacrificial template-based approaches are commonly used in synthesizing this tubular structure. Anodic aluminum anode (AAO), polycarbonate (PC) membrane, SiO_2_ and ZnO can be served as sacrificial templates in synthesizing metal oxides nanostructures. The synthesis process of SnO_2_ nanotubes usually includes three steps: (1) infiltration of reactants into the templates; (2) growth of SnO_2_ with designed shapes and morphology; (3) removal of the template.

**Fig. 2:**
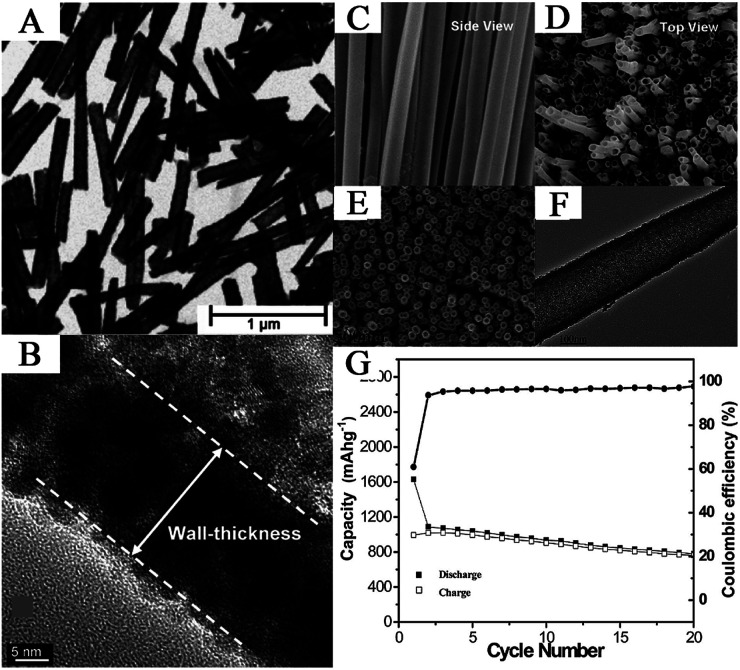
(A) TEM image of SnO_2_ nanotubes (adapted with permission from ref. 66 copyright 2006 Royal Society of Chemistry). (B) HRTEM image of SnO_2_ nanotubes for measuring thickness, (C) FESEM image of the side view, and (D) FESEM images of the top view of SnO_2_ nanotubes (adapted with permission from ref. 68 copyright 2005 American Chemical Society). (E) TEM image, (F) HRTEM image, and (G) discharge capacity *vs.* cycle number of SnO_2_ nanotube arrays on Ti substrates (adapted with permission from ref. 64 copyright 2011 American Chemical Society).

Early in 2006, Lai *et al.* reported the preparation of SnO_2_ nanotubes with a thickness of 10 nm and a length of about 0.4–1.4 μm *via* the electrodeposition method (Fig. 2A). They first electroplated the SnO_2_ nanoparticles on a gold electrode which was modified with a porous PC membrane. Then, the SnO_2_ particles were annealed at 650 °C in ambient conditions. The shape and size of the as-prepared nanotubes could be easily controlled by monitoring the charge passed.^66^ Meanwhile, Lai *et al.* also reported that the SnO_2_ nanotubes possessed better crystallinity and uniformity in terms of length and width.

The AAO and PC membrane-template methods have been widely used in the synthesis of metal oxide nanotubes. Wang *et al.* fabricated uniform polycrystalline SnO_2_ nanotubes *via* this template.^68^ They found that the diameter, thickness, length, and texture of nanotubes can be controlled with template structure, pristine particle size, and heating-rate temperature. As shown in Fig. 2B–D, nanotube walls, with a thickness of about 10–25 nm, were composed of abundant nanocrystallites of 6–15 nm. Furthermore, the SnO_2_ nanotube electrode showed superior charge/discharge performance compared to SnO_2_ nanoparticles. The specific capacity of the SnO_2_ nanotube electrode can be 525 mA h g^−1^ after 80 cycles.^68^ They summarized that the improved cycling performance resulted from the following factors, (1) cavities in the nanotubes which provide space and reduce the effect of volume change during lithiation/delithiation process; (2) SnO_2_ nanotubes provide more active sites for Li^+^ intercalation and deintercalation; and (3) compared to SnO_2_ nanoparticles, the SnO_2_ nanotubes are less movable and result in less agglomeration.^68^ Si and SiO_2_ have been commonly used in the synthesis of hollow nanostructures, including nanotubes. Morphology and shapes of SnO_2_ nanotubes can be well designed by controlling the shape of the Si templates and the hydrothermal conditions.^168^ Ye *et al.* also discovered that the shorter SnO_2_ nanotubes showed superior electrochemical performance, since the hollow structure in the short nanotubes can alleviate the volume changes.^147^ ZnO is another promising sacrificial template used for synthesizing SnO_2_ nanotube arrays as ZnO can be conveniently synthesized and removed. The as-prepared nanotubes exhibited a diameter and thickness of about 100–300 nm and about 10–20 nm, respectively, composed of nanoparticles with diameters of about 2–5 nm (Fig. 2E and F). The material also showed high capacity and improved cycling performance, *i.e.*, 750–800 mA h g^−1^ after 20 cycles at 0.1C, as shown in Fig. 2G.^64^

#### 2.1.3 SnO_2_ nanowires

Template-based methods have also been widely used to synthesize SnO_2_ nanowires. These sacrificial templates contribute to the development of mesoporous and hollow SnO_2_ nanowires.^63,169^ Kim *et al.* fabricated a SnO_2_ nanowire-based anode material for LIBs by using KIT-6 and SBA-15 SiO_2_ as hard templates. They first dissolved SnCl_4_·5H_2_O and the hard templates in DI water and stirred the mixture at room temperature until Sn^4+^ was adsorbed by templates. Then, the resulting composites were annealed, and the templates were removed from the composites using a NaOH solution. The as-obtained SnO_2_ nanowires had a diameter of 6 nm and a length greater than 3 μm, and the size of mesoporous SnO_2_ on the surface of the nanowire was 3.8 nm with a BET surface area of 160 m^2^ g^−1^. Such a porous and interconnected SnO_2_ nanowire structure showed better lithium storage capacity. The initial discharge and charge capacities of the nanowire anode were 1595 and 800 mA h g^−1^ at 0.2C, respectively, and the discharge capacity remained at 773 mA h g^−1^ after 50 cycles. These observations can be attributed to the mesopores which act as a buffer zone against SnO_2_ volume expansion.^71^

**Fig. 3:**
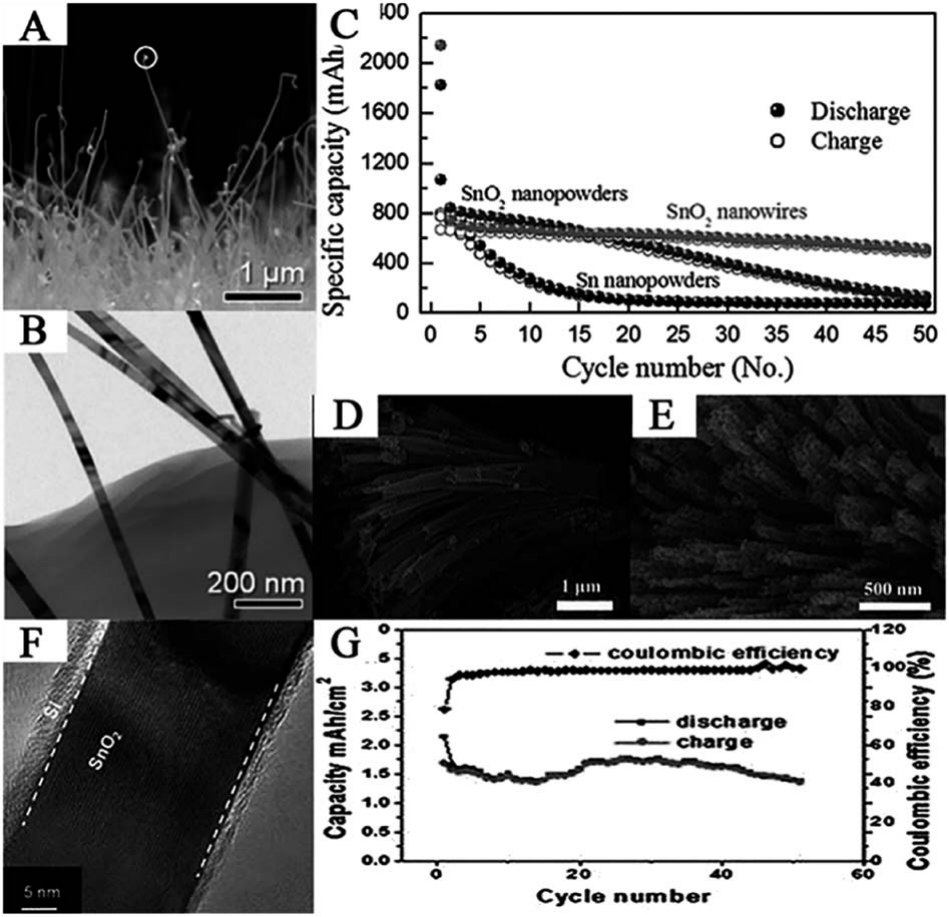
(A) Cross-sectional FESEM image, (B) TEM image, and (C) comparative cycling performance of the SnO_2_ nanowires (adapted with permission from ref. 72 copyright 2009 IOP Publishing). (D and E) SEM images of SnO_2_ porous nanowire bundles (adapted with permission from ref. 170 copyright 2011 Royal Society of Chemistry). (F) HRTEM image, and (G) cycling performance of the SnO_2_@Si nanowires (adapted with permission from ref. 73 copyright 2013 Royal Society of Chemistry).

Researchers have synthesized SnO_2_ nanowires *via* many methods. For example, Ko *et al.* synthesized SnO_2_ nanowires on the current collector *via* thermal evaporation at (600 °C). The as-prepared SnO_2_ nanowires exhibited a highly-ordered single-crystalline phase with a thin diameter of 40–50 nm and length of more than 1 μm (Fig. 3A and B). The SnO_2_ nanowire-based anode exhibited high specific discharge capacity and good cycling performance, *i.e.*, 2140 mA h g^−1^ at the first cycle and 510 mA h g^−1^ at the 50^th^ cycle at 1C (Fig. 3C).^72^ Ding *et al.* reported a facile strategy for the synthesis of SnO_2_ nanowire arrays using SBA-15 nanorods as a template by infiltrating molten SnCl_2_ into the channels of the SBA-15 nanorods followed by calcination and removal of the template.^69^ Han *et al.* investigated the synthesis of porous SnO_2_ nanowire bundles with a high yield *via* solution-based approaches. This hierarchical nanostructure is made up of SnO_2_ nanowires with an overall diameter of 80–120 nm and an average length of 6 μm (Fig. 3D). Moreover, the as-prepared nanowires have a highly porous structure composed of numerous nanocrystals.^170^ Ren *et al.* synthesized 3D hierarchical SnO_2_ nanowire arrays on carbon cloth by first, a CVD method for SnO_2_ nanowires, followed by a Plasma Enhanced-CVD (PECVD) method for Si thin film coating. The SnO_2_@Si nanowire arrays can directly serve as a flexible and binder-free anode for LIBs. In this unique structure, SnO_2_ nanowires act as a lithium storage material and a conductive matrix to support Si; in addition, the thin Si layer acts as a buffer for SnO_2_ to reduce the effect of volume changes (Fig. 3F). Anodes based on such novel hierarchical structures showed excellent electrochemical performance with discharge capacity of 2.13 mA h cm^−2^ in the first cycle and 1.386 mA h cm^−2^ after 50 cycles, *i.e.*, 65% of the first cycle (Fig. 3G).^73^ Thus, in this section, different synthesis methods for SnO_2_ nanowires, corresponding morphologies, and electrochemical properties have been presented, demonstrating that SnO_2_ nanowires are promising anode materials for LIBs.

### 2.2 2D nanomaterials

Due to the difficulty of synthesizing well-defined 2D SnO_2_ nanomaterials, obtaining a large variety of 2D SnO_2_ nanostructures is significantly harder than 1D and 3D nanomaterials. The anisotropic crystal growth of SnO_2_, in the formation process, limits the synthesis of 2D nanomaterials.^82^ In order to overcome this problem, *in situ* growth of SnO_2_ nanosheets on physical or chemical substrates has been widely investigated.^171,172^ Among these fabrication processes, SnO_2_ nanosheets grown on physical substrates, such as metal foam,^49,173–175^ foil,^77,154,176–178^ carbon cloth^78,179^ and graphite paper^180–183^ can be directly used as binder-free and current-collector free electrode in LIBs due to excellent conductive and supportive properties of such substrates. Meanwhile, some organic macromolecules can be used as chemical templates to fabricate SnO_2_ nanosheets. These substrates could provide a growth plane for SnO_2_ nanosheets and inhibit self-aggregation during the lithiation/delithiation processes.^184^ Moreover, SnO_2_ nanosheets have a tendency to assemble into complex structures, such as nanosheet arrays, clusters, and spheres, which usually exhibit superior lithium storage capacities due to their highly interconnected and hierarchical nanostructures.^184–187^

**Fig. 4:**
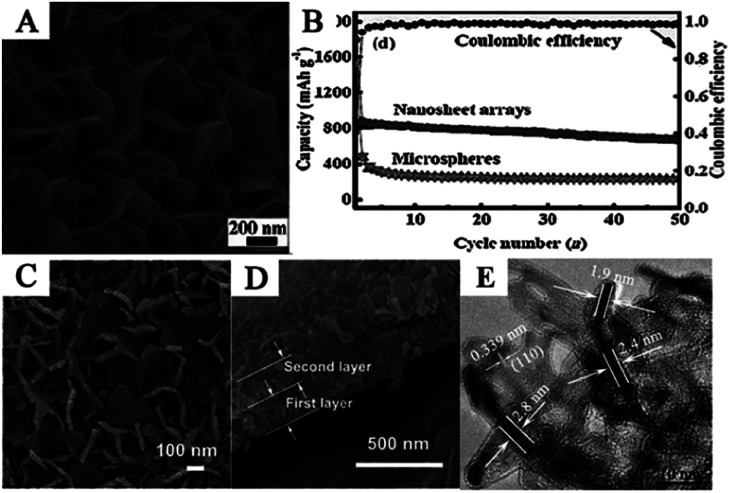
(A) SEM image, and (B) cycling performance of hierarchical SnO_2_ nanosheet arrays on nickel foam (adapted with permission from ref. 79 copyright 2014 Wiley). (C) Top view, and (D) cross-sectional FESEM images of SnO_2_ nanosheets double layer on Ti foil (adapted with permission from ref. 77 copyright 2014 Royal Society of Chemistry). (E) HRTEM image of ultrathin SnO_2_ nanosheets of less than 5 nm (adapted the permission from ref. 80 copyright 2015 American Chemical Society).

Zhao *et al.* fabricated SnO_2_ nanosheet arrays on Ni foam with a thickness of 20 nm and a length of 500 nm (Fig. 4A) *via* a hydrothermal method. Nickel foam, *i.e.*, a 3D macroporous conductive network, was used as a supportive substrate to fabricate SnO_2_ nanosheet arrays, which can be directly used as a binder-free anode in LIBs. Due to the high electroactive surface area, ultrathin sheets, and shorter electron transport pathways, the nanosheets exhibited excellent electrochemical properties. The novel anode showed an initial discharge capacity of approximately 1800 mA h g^−1^ and remained at 674.9 mA h g^−1^ after 50 cycles at 0.5C (Fig. 4B).^79^ Moreover, interconnected single and double layer SnO_2_ nanosheets were also fabricated on three different conductive substrates, *i.e.*, Ti, Cu foil, and flexible graphite paper, as integrated binder-free electrodes for LIBs. The nanosheets were interconnected with each other to form a hierarchical network, and the thickness of a single SnO_2_ nanosheet layer was about 250 nm (Fig. 4C and D). The electrodes delivered high specific capacity, excellent cycling stability, and good rate capability.^77,79^ Zhu *et al.* reported an ultra-rapid, low-cost, and simple microwave-assisted synthesis of ultrathin SnO_2_ nanosheets with a thickness of less than 5 nm (Fig. 4E). The ultrathin SnO_2_ nanosheets exhibited significantly enhanced electrochemical lithium storage properties with a high reversible capacity of 757.6 mA h g^−1^ at a current density of 200 mA g^−1^ up to 40 cycles. The ultrathin 2D nanosheets can significantly reduce the ion diffusion paths and allow faster phase transitions; furthermore, the sufficient external surface interspace and porous configuration successfully accommodate the large volume changes.^77,78,80^

**Fig. 5:**
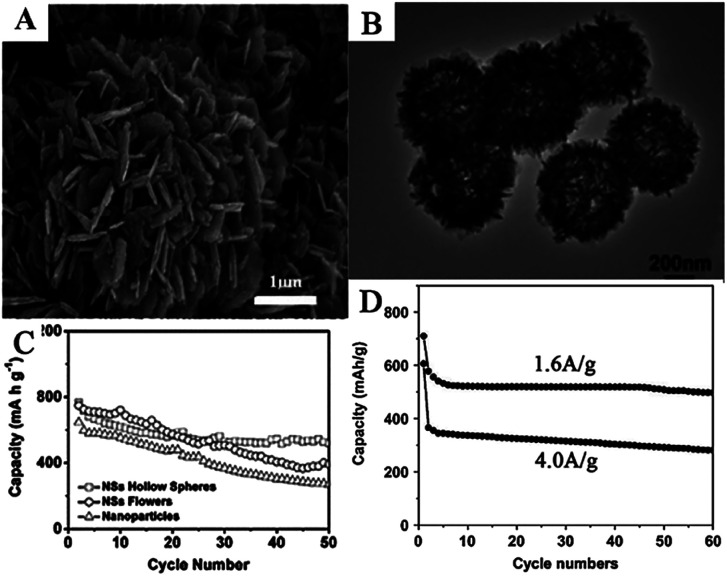
(A) SEM images of flower-like SnO_2_ nanosheets (adapted with permission from ref. 78 copyright 2014 Elsevier). (B) TEM image, and (C) cycling performance of SnO_2_ nanosheets hollow spheres (adapted the permission from ref. 87 copyright 2011 Royal Society of Chemistry). (D) Cycling performance of SnO_2_ nanosheets at 1.6 A g^−1^ and 4.0 A g^−1^ (adapted the permission from ref. 81 copyright 2017 Elsevier).

Many complex hierarchical SnO_2_ nanostructures were also assembled by 2D SnO_2_ nanosheets. Flower-like SnO_2_ assembled by many SnO_2_ nanosheets, with an average thickness of 100 nm, have been synthesized on a flexible carbon cloth using CVD at 750 °C.^78^ SEM images (Fig. 5A) show that as-prepared flower-like SnO_2_ nanostructures were formed by an assembly of numerous nanosheets with a thickness of 100 nm. Ding *et al.* synthesized hollow spheres assembled from SnO_2_ nanosheets (Fig. 5B), using sulfonated polystyrene hollow spheres (sPSHSs) as a template; the surface of the sPSHS is covered with –SO_3_– functional groups. Therefore, the Sn^2+^ ions can easily interact with these templates *via* electrostatic forces and subsequently grow into SnO_2_ nanosheets, assisted by mercaptoacetic acid. Moreover, sPSHSs templates are beneficial for minimizing the gas outflux during the template removal process, which helps the retention of the final structure.^188–191^ The as-prepared samples showed a superior cycling capacity retention compared to other SnO_2_ nanoflowers assembled by SnO_2_ nanosheets as well as SnO_2_ nanoparticles, indicating the positive effect of the unique nanostructure (Fig. 5C).^87^ Wei *et al.* fabricated nanoporous SnO_2_ nanosheets *via* a simple one-step ultrasonic-assisted chemical precipitation strategy with polyvinylpyrrolidone (PVP) as a soft template. Due to the porous nanosheet structure, it exhibited high capacity, *i.e.*, an initial discharge capacity of 2231 mA h g^−1^ at 0.2 A g^−1^, and excellent cycling performance, *i.e.*, an initial discharge capacity of 710 mA h g^−1^ and 606 mA g^−1^ at 1.6 A g^−1^ and 4 A g^−1^, respectively, which remained at 497 mA h g^−1^ and 280 mA h g^−1^ after 60 cycles, respectively (Fig. 5D).^81^

### 2.3 3D nanomaterials

In recent years, 3D SnO_2_ structures have attracted significant attention.^86^ Various 3D nanostructures have been synthesized, such as hollow spheres^91,192–194^ and microboxes.^195–197^ Furthermore, many new synthetic methods to prepare SnO_2_ hollow structures, including Ostwald ripening,^198–200^ Kirkendall effect,^201–204^ removable templates^195^ and chemically induced self-assembly^192,205^ have been reported.

**Fig. 6:**
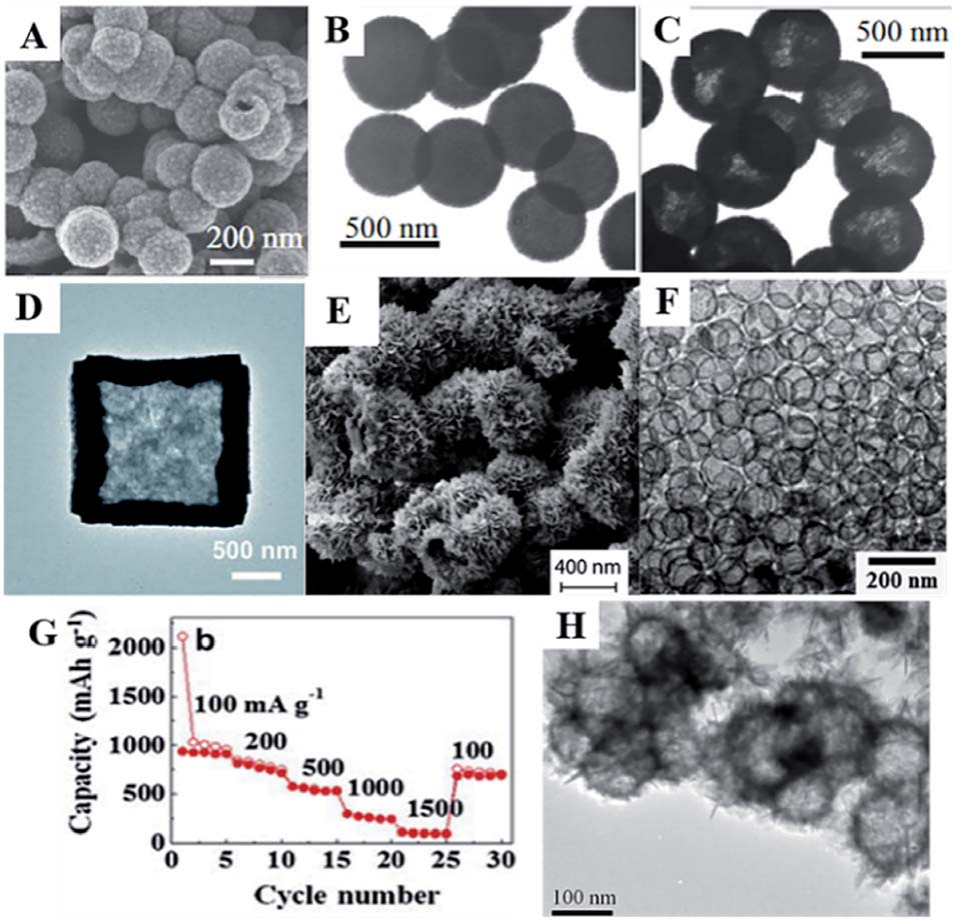
(A) FESEM images of SnO_2_ hollow nanosphere prepared in the solvent of EtOH : H_2_O ratio of 1 : 1. (B) TEM images of SnO_2_ hollow nanosphere prepared with the value of EtOH in the solvent of EtOH–H_2_O = 37.5%. (C) TEM images of SnO_2_ hollow nanosphere prepared with the value of EtOH in the solvent EtOH–H_2_O = 37.5% and 0.1 M urea (adapted the permission from ref. 86 copyright 2006 WILEY). (D) TEM images of SnO_2_ microboxes (adapted the permission from ref. 195 copyright 2016 Royal Society of Chemistry). (E) SEM images of SnO_2_ hierarchical hollow microspheres (adapted the permission from ref. 205 copyright 2017 Royal Society of Chemistry). (F) TEM images, and (G) rate performance of SnO_2_ hollow spheres (adapted with permission from ref. 91 copyright 2012 Elsevier). (H) TEM image of the hierarchical SnO_2_ (adapted with permission from ref. 210 copyright 2010 American Chemical Society).

In 2006, Lou *et al.* investigated a one-pot template-free synthesis of hollow SnO_2_ nanostructures, based on an unusual inside-out Ostwald ripening mechanism. The synthesis process was performed in an ethanol–water (EtOH–H_2_O) mixed solvent with K_2_SnO_3_ as the precursor, followed by a hydrothermal treatment. Lou *et al.* discovered that the concentration of the precursor and the ratio of EtOH in the mixed solvent determined both the particle size and morphology of the product. Moreover, the addition of urea or thiourea in the synthetic mixtures was also found to increase the product yield, morphological yield to nearly 100%, and a well-dispersed monodispersity (Fig. 6A–C). Such hollow SnO_2_ nanosphere-based anode material exhibited high discharge capacity and good cycling performance, *i.e.*, an initial discharge capacity of 1140 mA h g^−1^, which is comparable to the theoretical capacity of graphite after more than 40 cycles.^86^

Porous hollow SnO_2_ micro-boxes have been synthesized *via* a selective leaching strategy using ZnSn(OH)_6_ as the precursor. ZnSn(OH)_6_ micro-boxes were first formed through a modified one-pot co-precipitation method and subsequently, the Zn species were removed *via* a selective leaching strategy. The TEM image (Fig. 6D) shows that the thickness of the shell was about 100 nm.^195^ Hu *et al.* used a template-and additive-free hydrothermal route to prepare a uniquely shaped SnO_2_ material that comprised of a hollow spherical morphology with uniform diameter and very thin petal-like nanosheets grown perpendicularly on the surface of the spheres, resembling a “chestnut cupule” (Fig. 6E). In contrast to conventional SnO_2_ materials, this unique morphology significantly improved the storage capacity and cycling performance of SnO_2_ as an anode material for lithium and sodium ion batteries.^206^

In addition, 3D hierarchical SnO_2_ nanomaterials can be assembled by 0D, 1D, and 2D SnO_2_ nanomaterials. For example, 0D crystalline SnO_2_ nanoparticles were successfully assembled into a high-order nanostructure of hollow core–shell. First, SnCl_4_ was dissolved in a mixture of DI water and ethanol to form a homogeneous solution which was heated to 180 °C for 24 h. Then, the collected sediments were calcined in air to remove carbon by oxidation. FESEM showed that the meso-spheres had an overall dimension of 1–3 μm, and the surface of the spheres was formed by small aggregated SnO_2_ nanoparticles (11 nm). The shell was estimated to be approximately 200 nm in thickness. This unique SnO_2_ nanostructure could store an exceedingly large amount of Li^+^ and cycled well for a pure phase SnO_2_ anode.^207–209^ Hollow urchin-like SnO_2_ nanospheres have been fabricated using ultrathin nanorods *via* a solvothermal route. The diameters of urchin-like nanospheres and nanorods are about 300 and 100 nm, respectively. The as-obtained hollow urchin-like SnO_2_ nanospheres with ultrathin 1D nanorods exhibited high capacity and excellent rate discharging performance. The 1^st^, 2^nd^, 20^th^, and 50^th^ discharge capacities were 1881 mA h g^−1^, 1090 mA h g^−1^, 781 mA h g^−1^, and 719 mA h g^−1^, respectively, at a current density of 100 mA g^−1^. Upon changing the discharge–charge rates to 0.2, 0.4, 0.8, 1, 2, and 0.4C, the capacities of urchin-like SnO_2_ were maintained at 815, 687, 601, 446, 282, and 520 mA h g^−1^, respectively, while for commercial SnO_2_, the capacities were only 828, 677, 512, 386, 246, 133, and 375 mA h g^−1^, respectively. The retention of the reversible capacity of the hollow nanosphere electrodes was better than that of commercial SnO_2_ samples.^210^ Furthermore, SnO_2_ hollow nanospheres can also be synthesized by sol–gel methods.^91^ The size of the hollow spheres is controlled by using different-sized templates. As-prepared SnO_2_ shells are almost amorphous and exhibit a rutile phase after annealing at 600 °C. The size of the SnO_2_ hollow spheres ranges from 25 to 100 nm (Fig. 6F), and the thickness of the shell is constantly 5 nm despite the size of the hollow spheres. Due to the nanosized hollow sphere and thin shell thickness, SnO_2_ hollow spheres show excellent electrochemical performance. The smallest hollow sphere of SnO_2_ (25 nm) exhibited a high reversible capacity of 750 mA h g^−1^ as well as good rate performance, *i.e.*, 700 mA h g^−1^ at 0.2 A g^−1^ and 530 mA h g^−1^ at 0.5 A g^−1^ (Fig. 6G).^91^ Hierarchical SnO_2_ hollow nanostructures can also be assembled by 2D nanosheets. As shown in Fig. 6H, the hierarchical SnO_2_ shows high capacities and excellent cycle performance as an anode material for LIBs. The improved electrochemical properties could be ascribed to the large surface area, enhanced structure stability, and short diffusion length for both lithium ions and electrons.^211^

## 3. Composites of SnO_2_ and carbonaceous materials

### 3.1 SnO_2_ with carbon nanotubes (CNTs)

Nanocomposites of SnO_2_ and CNTs reportedly exhibit improved lithium storage capacities compared to pure SnO_2_ materials.^212^ This can be attributed to the flexible nature and superior conductivity of the CNTs, which alleviates the internal stress caused during the charge/discharge process^213–215^ and enhances the electron transportation. With increased conductivity and surface area, such nanocomposites show enhanced lithium storage capability.^216,217^

Wen *et al.* reported an *in situ* synthesis of mesoporous SnO_2_ on the surface of multi-walled CNTs (MWCNTs) through a hydrothermal method utilizing CTAB as the structure-directing agent. The MWCNTs/SnO_2_ hybrid electrodes showed great electrochemical performance and cycling stability. This can be attributed to the synergistic effects of the unique combination of properties including their one-dimensional hollow structure, high-strength with flexibility, excellent electric conductivity, and large surface area, which helped alleviate the effect of volume expansion, shorten the distance of Li^+^ diffusion, and contribute to the transmission of electrons.^100,218–221^

**Fig. 7:**
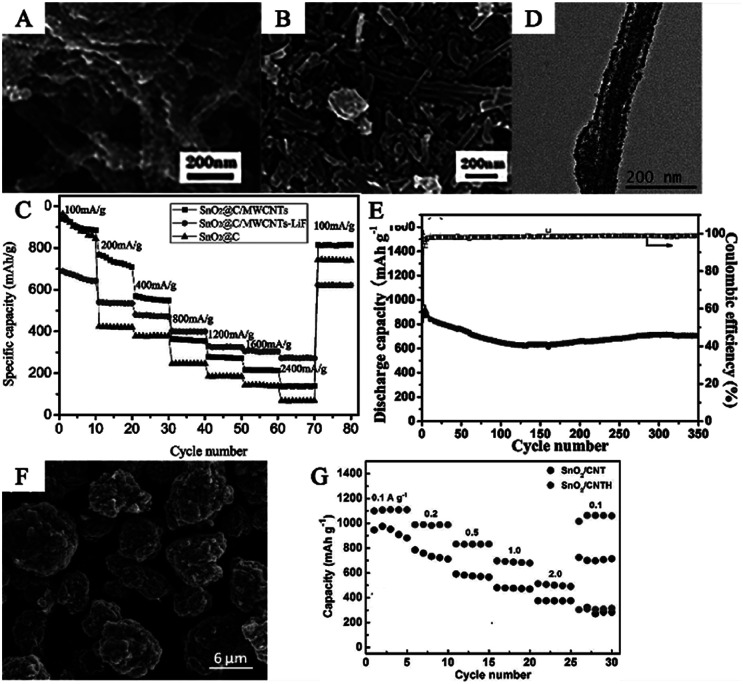
(A) FESEM image of SnO_2_/CNT composites prepared with MSn : C = 0.3 : 1 (adapted with permission from ref. 221 copyright 2009 Elsevier). (B) SEM image, and (C) rate performance of SnO_2_@C/MWCNTs-LiF (adapted with permission from ref. 224 copyright 2019 Elsevier). (D) TEM image and (E) cycling performance of CNT@void@SnO_2_@C composite (adapted the permission from ref. 225 copyright 2015 Elsevier). (F) SEM image, and (G) rate performance of SnO_2_/CNTH composites (adapted with permission from ref. 226 copyright 2019 American Chemical Society).

In another hydrothermal system, Du *et al.* synthesized SnO_2_/MWCNT composites by a simple solvothermal method and subsequent heat treatment at 360 °C with SnCl_2_ and CNTs as reactants. The distribution of SnO_2_ nanocrystals can be controlled by changing the molar ratio of Sn^2+^ and CNTs in the precursor. For SnO_2_/MWCNTs composites prepared with a molar ratio of Sn : C = 0.3 : 1, a uniform layer of SnO_2_ nanocrystals, with a crystal size of about 5 nm, was deposited on the surface of the MWCNTs (Fig. 7A).^222^ These composites showed very stable cycling retention, of up to 100 cycles, because of nanosized materials and the introduction of CNTs.^201,223^ Jin *et al.* prepared SnO_2_/MWCNTs electrodes *via* a hydrothermal method at 150 °C for 24 h. The SnO_2_ nanoparticles with diameters of less than 3 nm were uniformly loaded onto the surface of MWCNTs. The MWCNT/SnO_2_ nanocomposites exhibited a high reversible capacity of 420 mA h g^−1^ even after 100 cycles.^137,224^

In order to further improve electrochemical properties of the SnO_2_/CNTs hybrid nanomaterials, many groups have synthesized various SnO_2_/CNTs-based anode materials. For example, Liang *et al.* synthesized SnO_2_@C/MWCNTs-lithium fluoride (LiF) composite nanomaterials (Fig. 7B). Carbon-coated SnO_2_ (SnO_2_@C) was prepared by a spray drying method, with water-soluble asphalt as the carbon source and MWCNTs as the conductive agent. The conductivity was significantly enhanced, and the extent of volume expansion of the SnO_2_ was reduced. Compared to SnO_2_/CNTs, the presence of LiF enhanced the stability of the SEI film and improved the coulombic efficiency and capacity retention rate of the electrode. After 200 cycles, the SnO_2_@C/MWCNTs-LiF anode still maintained 70.1% of the capacity retention rate and the specific capacity was held at 274 mA h g^−1^ at 2400 mA g^−1^, compared with 136 mA h g^−1^ for the SnO_2_@C/MWCNTs anode (Fig. 7C).^225^ Tian *et al.* fabricated a tube-in-tube nanostructure, denoted as CNT@void@SnO_2_@C, by a facile hydrothermal method and subsequent carbonation with polysaccharide as the carbon source, SiO_2_ as the sacrificial template, and NH_4_F as the etchant. The CNT@void@SnO_2_@C exhibited one-dimensional nanostructures with average diameters of about 100–150 nm and hollow structures filled with abundant voids (Fig. 7D). Tian *et al.* interpreted the formation mechanism as follows: (1) the crystallization and deposition of SnO_2_ could occur prior to the polycondensation of glucose; (2) the formation rate of SnO_2_@polysaccharide was faster than the etching rate of SiO_2_ by NH_4_F due to the slow etching process of SiO_2_ by NH_4_F solution under hydrothermal condition; and (3) the large void-space between the SnO_2_@polysaccharide and CNT, formed after the SiO_2_ coating layer, was etched away completely. The CNT@void@SnO_2_@C exhibited good electrochemical properties, delivering a reversible capacity of 702.5 mA h g^−1^ at 200 mA g^−1^ even after 350 cycles (Fig. 7E). This indicated that the unique tube-in-tube nanostructure, as well as the one-dimensional void space, which formed between the inner CNT and outer SnO_2_@C nanotubes, contributed significantly to the electrochemical performance.^226^ Liu *et al.* synthesized ultrafine SnO_2_ (6–7 nm)/carbon nanotube hairball (SnO_2_/CNTH) composites with a 3D hierarchical structure (Fig. 7F), which was prepared by spray drying and a solvothermal method. Fig. 7G shows that SnO_2_/CNTH exhibited superior electrochemical performance and improved the lithium storage capacity compared to conventional SnO_2_/CNT. The improved electrochemical performance can be attributed to the increased conductivity and enhanced electrode reactivity due to the 3D hierarchical cross-linked structure. This structure can also address the large volume changes upon cycling.^227^

### 3.2 SnO_2_ with amorphous carbon

Hydrothermal treatment followed by carbonization is the most common method for synthesizing SnO_2_/amorphous carbon nanomaterials.^228–230^ There are mainly two synthesis routes of SnO_2_/amorphous carbon; one is to treat Sn^2+^ or Sn^4+^ salt with the precursor of carbon,^231–236^ and the other is to deposit a carbon layer on the as-prepared SnO_2_ nanostructures.^237,238^ Conventional amorphous carbon can be derived from glucose,^239,240^ sucrose^241,242^ and many organic compounds.^243^ The morphology and shapes of the nanostructures can be adjusted by the hydrothermal and annealing conditions.

**Fig. 8:**
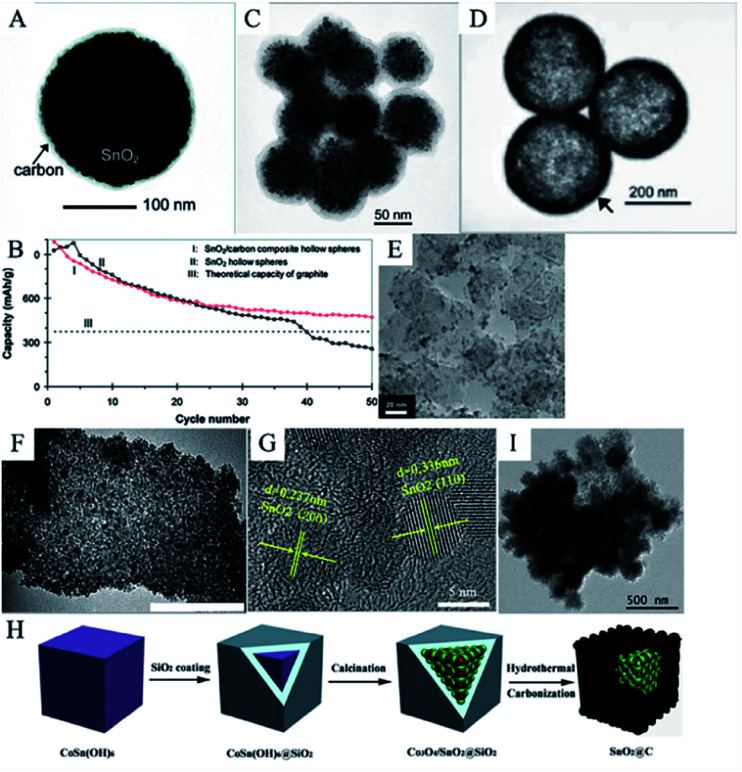
(A) TEM image, and (B) cycling performance of SnO_2_/carbon composites hollow spheres (adapted with permission from ref. 99 copyright 2008 American Chemistry Society). (C) TEM image of carbon-coated SnO_2_ nanocolloids obtained after carbonization at 550 °C (adapted with permission from ref. 239 copyright 2009 American Chemical Society). (D) TEM image of SnO_2_@carbon hollow nanospheres (adapted with permission from ref. 240 copyright 2009 Wiley). (E) TEM image of nanosized SnO_2_/carbon composites (adapted with permission from ref. 248 copyright 2009 Elsevier). (F) TEM image, and (G) HRETM image of SnO_2_/RHPC composite materials (adapted with permission from ref. 231 copyright 2019 Elsevier). (H) The fabrication schematic of SnO_2_@non-smooth carbon. (I) TEM image of SnO_2_ quasi-nanocubes@non-smooth carbon (adapted with permission from ref. 255 copyright 2019 Elsevier).

Various SnO_2_/C nanomaterials have been synthesized in recent years, such as carbon-coated SnO_2_ NPs,^244–247^ carbon-coated SnO_2_ nanorods,^248,249^ carbon-coated SnO_2_ nanowires^151,217^ and carbon-coated SnO_2_ nanotubes.^57^ Different structures and morphologies of SnO_2_ are known to lead to different electrochemical performance. In 2008, Lou *et al.* prepared SnO_2_/C composite hollow spheres. The mesoporous SnO_2_ hollow spheres were embedded in 3D carbon networks (Fig. 8A). The carbon networks act as a physical buffering cushion for the intrinsic large volume change and electronically conducting pathways. Compared to SnO_2_ hollow spheres and graphite, these SnO_2_/carbon hollow spheres were able to deliver a reversible lithium storage capacity of 473 mA h g^−1^ after 50 cycles (Fig. 8B).^99^ In 2009, Lou *et al.* also synthesized a thin layer of carbon-coated SnO_2_ nano-colloids (Fig. 8C) and coaxial SnO_2_@carbon hollow nanospheres (Fig. 8D) by a simple hydrothermal method followed by carbonization; both exhibited improved electrochemical performance.^57,250^ Courtel *et al.* reported an *in situ* synthesis of SnO_2_ nanoparticles (5–10 nm)/carbon composite materials using the polyol method by oxidizing SnCl_2_·2H_2_O in the presence of a carbon matrix. The TEM image (Fig. 8E) show that the SnO_2_ nanoparticles were uniformly embedded in the carbon matrix. Based on the nanostructure, the as-obtained composites showed an improved lithium storage capacity of 370 mA h g^−1^ at 200 mA g^−1^ and a lower capacity fading compared to commercial SnO_2_ (50 nm).^251^

While the morphology and structure of SnO_2_ influence the electrochemical properties of the SnO_2_/C composites, different carbonaceous materials also influence the performance of the composite. Xu *et al.* prepared composites of SnO_2_/ordered mesoporous carbon (SnO_2_/OMC) through a hydrolysis process. OMC, a novel kind of carbon material, has been widely used in LIBs due to its large surface area, high conductivity, and highly porous structure; it promotes the diffusion of lithium ions and electrolyte. The SnO_2_/OMC composites delivered a good cycling performance with a reversible capacity of 395.6 mA h g^−1^ for up to 50 cycles.^252^ Shi *et al.* synthesized SnO_2_/rice-husk-based porous carbon composites (RHPC) *via* a simple melt-impregnation method with a heat treatment route. RHPC can be easily and cheaply obtained by convenient carbonization and activation of rice husk. The TEM images (Fig. 8F and G) show that the SnO_2_ nanoparticles with the average size of 4 nm can be loaded on the RHPC matrix. The cycling measurements showed that the discharge capacity of the RHPC/SnO_2_ anode at the current density of 100 mA g^−1^, at the 50^th^ cycle, was 550 mA h g^−1^, which demonstrated that in contrast to pure SnO_2_ anodes, the cycling performance of the RHPC/SnO_2_ anode was remarkably enhanced by the introduction of the RHPC matrix. It also demonstrates that biomass-sourced carbonaceous materials like RHPC^253–255^ have promising applications in LIBs due to their low cost and porous structure.^233^ Tian *et al.* fabricated non-smooth carbon-coated SnO_2_ quasi-nanocubes. Generally, in SnO_2_/C nanocomposites, nanostructured SnO_2_ is usually coated with smooth carbon, which is easily fabricated from organically sourced carbon *via* hydrothermal or CVD methods.^90,256^ However, it is believed that SnO_2_-coated 3D non-smooth carbon usually shows better lithium storage properties owing to the substantial free space and larger surface area of the 3D structure.^257^ In this work, Tian *et al.* synthesized the hybrid nanostructures *via* multiple hydrothermal and calcination methods (Fig. 8H). As shown by the TEM images (Fig. 8I), the porous SnO_2_ quasi-nanocubes were coated by a carbon layer with a rough surface. The introduction of 3D non-smooth carbon can reduce the transmission length of electrons and Li^+^ and increase the electrochemical reaction sites. Based on such 3D porous structures, the SnO_2_@C anode displayed extraordinary cycling performance and outstanding rate capability, maintaining a capacity of 1089.5 mA h g^−1^ at 200 mA g^−1^, even after 400 cycles, as well as 479.2 mA h g^−1^ at 3000 mA g^−1^.^257^

It is worth mentioning that even though the amorphous carbon layer can enhance the cycling stability and conductivity of the composites, it has a relatively low lithium storage capacity compared to SnO_2_. Therefore, the ratio of the carbon layer and the SnO_2_ nanomaterial determines the capacity and cycle life of the composites.

### 3.3 SnO_2_/graphene

As an important 2D carbon material, graphene has quickly gained importance in material science in recent years. Due to its excellent mechanical properties and superior conductivity, graphene has been widely studied in LIBs.^258–260^ Compared to CNTs and amorphous carbon, graphene exhibits higher specific discharge capacity, high surface area, good mechanical properties, and high chemical stability;^261–263^ thus, SnO_2_/graphene, as an anode material in LIBs, shows better electrochemical performance.^264–268^

**Fig. 9:**
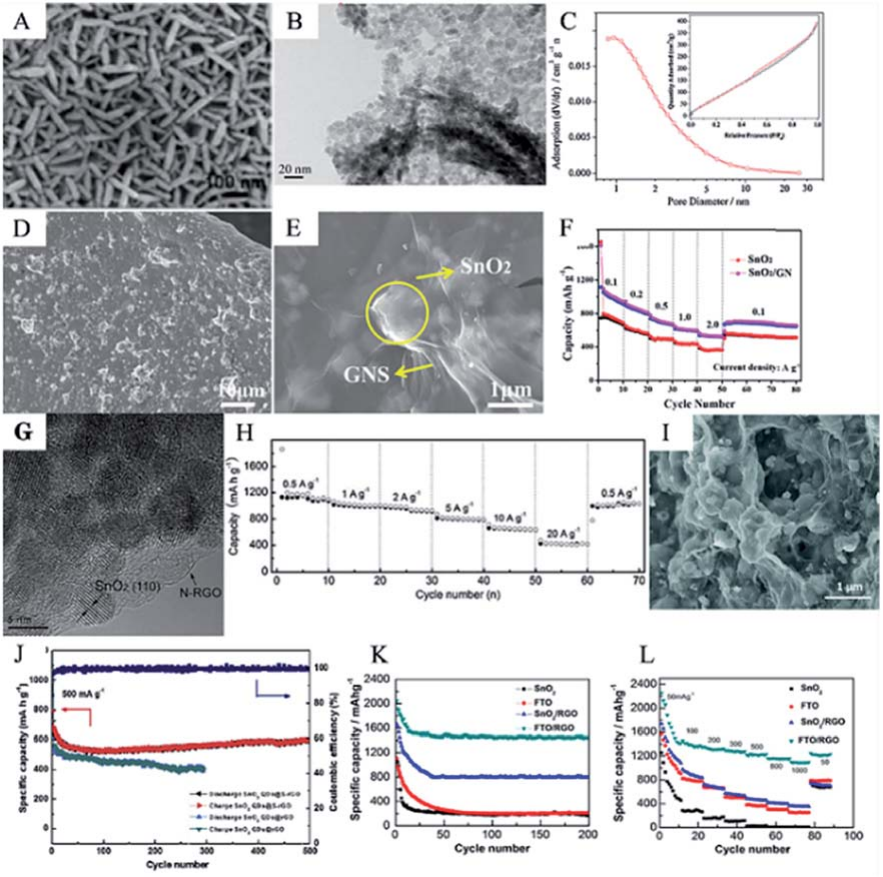
(A) SEM image of SnO_2_ nanosheets/graphene (adapted with permission from ref. 170 copyright 2011 Royal Society of Chemistry). (B) TEM image and (C) nitrogen adsorption–desorption isotherms of SnO_2_ nanoparticles/graphene (adapted with permission from ref. 169 copyright 2017 Elsevier). (D and E) SEM images and (F) rate performance of SnO_2_/GN on Ni foam (adapted with permission from ref. 267 copyright 2018 Elsevier). (G) HRTEM image and (H) rate performance of SnO_2_NC@N-RGO (adapted with permission from ref. 173 copyright 2013 Wiley). (I) SEM images of SnO_2−*x*_/N-rGO (adapted with permission from ref. 280 copyright 2018 Royal Society of Chemistry). (J) Cycling stability of SnO_2_QDs@S-rGO (adapted with permission from ref. 277 copyright 2018 Elsevier). (K) Cycling performance and (L) rate performance of FTO/rGO (adapted with permission from ref. 109 copyright 2015 American Chemical Society).

Graphene can be used as a supporting substrate for the synthesis of hierarchical SnO_2_ nanostructures. For example, Ding *et al.* fabricated 2D SnO_2_ nanosheets grown on a graphene oxide (GO) support *via* a facile hydrothermal method. The SnO_2_ nanosheets were uniformly embedded in the GO support, approximately 100 nm in length and 5–10 nm in thickness (Fig. 9A). This unique SnO_2_/GO hybrid structure exhibited enhanced lithium storage properties with high reversible capacities, *i.e.*, an initial discharge capacity of 1666 mA h g^−1^, and good cycling performance, *i.e.*, 518 mA h g^−1^ after 50 cycles at 400 mA h g^−1^.^171^ Additionally, due to its mechanical properties and electronic conductivity, graphene can serve as the carbon matrix to accommodate the SnO_2_ nanoparticles. In 2017, Shi *et al.* investigated a facile microwave-assisted hydrothermal method to synthesize a composite of SnO_2_ and graphene, which took only 30 min and did not require any chelating agents. As shown by TEM images (Fig. 9B) and the nitrogen adsorption–desorption isotherms (Fig. 9C), Shi *et al.* found that ultra-small SnO_2_ nanoparticles were well dispersed on the surface of the graphene, with an average particle size of about 3–8 nm. It also showed superior lithium storage capability. The charge/discharge capacity of this material was 969.4/978.6 mA h g^−1^ after 100 cycles at 200 mA g^−1^.^269^ Binder-free multilayered SnO_2_/graphene on Ni foam was fabricated *via* a dip-coating method. SnO_2_ nanoparticles and grapheme were alternatively coated on to the Ni foam to obtain a sandwich-like structure. As shown in SEM images in Fig. 9D and E, SnO_2_ nanoparticles were uniformly distributed on the surface of the Ni foam and wrapped tightly in GN. Such a multilayered nanostructure showed superior electrochemical performance due to the following factors: (1) 3D porous Ni foam serves as a conductive network and binder-free current which is beneficial for electron and ion diffusion; (2) the graphene layer improves the conductivity of anode material and buffers the SnO_2_ volume changes; and (3) the extent of volume change of the SnO_2_ nanoparticles is lower than that of bulk SnO_2_. Owing to such porous Ni foam frameworks and sandwich-like structures, the SnO_2_/graphene composites exhibited good rate performance and excellent cycling stability. High capacities, *i.e.*, 708 and 609 mA h g^−1^ were achieved at current densities 1 and 2 A g^−1^, respectively (Fig. 9F). Furthermore, the SnO_2_/GN electrode delivered a high capacity of 757 mA h g^−1^ after 500 cycles at 1 A g^−1^.^174^

Moreover, it is worth mentioning that atom-doped graphene can also be used as a carbon matrix for SnO_2_, which effectively enhances the electrochemical performance of the composites. Heteroatoms in graphene can act as anchor sites that prevent aggregation and exfoliation of the SnO_2_ anchored on graphene; this helps improve the cycling stability of such anode materials.^270–274^ Liu *et al.* synthesized SnO_2_ nanoparticles anchored on chlorinated graphene (SnO_2_@rGO-Cl) as binder-free electrodes that exhibit a long cycling life, of up to 400 cycles, with a discharge capacity of 1008 mA h g^−1^*via* a facile strategy using a one-step heat treatment at low temperature.^275^ Liu *et al.* found that Cl-doping can enhance the electrical conductivity of graphene and the Cl–Sn bonds can prevent the exfoliation of SnO_2_ nanoparticles during the charge/discharge process; thus, improving the electrochemical properties of SnO_2_-based hybrid nanomaterials. SnO_2_/nitrogen-doped graphene (N-rGO) was also applied as an anode in LIBs. It is believed that N-doped carbonaceous materials enhance the electronic conductivity and SEI film stability.^276–278^ Zhou *et al.* fabricated SnO_2_@N-rGO *via* a hydrazine monohydrate vapor reduction approach for anchoring SnO_2_ nanocrystals uniformly into N-rGO (Fig. 9G). Due to the bond formed between SnO_2_ and graphene, and the void pores dispersed in N-rGO, the as-prepared hybrid materials displayed superior mechanical properties and lithium storage capacity. SnO_2_@N-rGO anode showed a reversible charge capacity of 1346 mA h g^−1^ after 500 cycles; furthermore, as the current density increased from 0.5 to 1, 2, 5, 10, and 20 A g^−1^, their discharge capacity varied from 1074 to 994, 915, 782, 631, and 417 mA h g^−1^, respectively (Fig. 9H).^279^ Wu *et al.* synthesized the SnO_2−*x*_/N-rGO hybrid material through electrostatic adsorption-induced self-assembly together with a thermal reduction process. This treatment induced the generation of the oxygen vacancies on the surface of SnO_2_ hollow nanospheres; thus, building up a long-range and bi-continuous transfer channel for rapid electron and ion transport. Meanwhile, SnO_2−*x*_ hollow spheres are well-wrapped by graphene sheets; thus, enhancing the conductivity of the anode material (Fig. 9I). Due to these structural advantages, the as-obtained SnO_2−*x*_/N-rGO electrode exhibited excellent robust cycling stability, *i.e.*, about 912 mA h g^−1^ after 500 cycles at 0.5 A g^−1^ and 652 mA h g^−1^ after 200 cycles at 1 A g^−1^, and superior rate capability, *i.e.*, 309 mA h g^−1^ at 10 A g^−1^.^280^ Sulfur-doped graphene (S-rGO) also proved to be a feasible anode material for LIBs.^281^ Compared to the C atom, the S atom has a larger volume and lower electronegativity, which is beneficial for the diffusion of Li^+^ and electrons. For instance, Wu *et al.* successfully loaded SnO_2_ quantum dots (QDs) on sulfur-doped reduced graphene oxide (S-rGO), and it exhibited excellent lithium storage with a high specific capacity of 897 mA h g^−1^ and a long cycling stability with 88% capacity retention after 500 cycles (Fig. 9J).^282^ The abovementioned results demonstrate that heteroatoms can tailor the electronic structure of carbon and create topological defects in the carbon lattice.^281,283^

Apart from doped-graphene, atom-doped SnO_2_, such as fluorine-doped tin oxide^138^ and antimony-doped tin oxide^122^ can also be used with pristine graphene as an anode material.^284–287^ For example, Xu *et al.* has successfully fabricated composites of fluorine-doped tin oxide (FTO) and rGO from a colloidal solution containing FTO nanocrystals and rGO by a hydrothermal treatment; the FTO nanocrystals were tightly embedded in the RGO nanosheets. As an anode material, the FTO/RGO composite showed high structural stability during the lithiation and delithiation processes. The conductive FTO nanocrystals help form stable and thin SEI films. Moreover, the FTO/RGO composite retains a discharge capacity as high as 1439 mA h g^−1^ after 200 cycles at 100 mA g^−1^ (Fig. 9J), and 1148 mA h g^−1^ at 1000 mA g^−1^ (Fig. 9K).^109^

### 3.4 Complex SnO_2_/C nanostructure

Compared to 1D or 2D carbonaceous materials, complex 3D carbonaceous matrices usually show better electrochemical properties due to their high specific surface area and porous structure, which is beneficial in suppressing volume changes.^288^ In recent years, various SnO_2_/C complex hybrid nanomaterials have been reported, such as the combination of CNTs and amorphous carbon,^288^ and CNTs and graphene.^283,289,290^ These complex hierarchical SnO_2_/C nanostructures often show superior electrochemical properties, making them an important research subject.

**Fig. 10:**
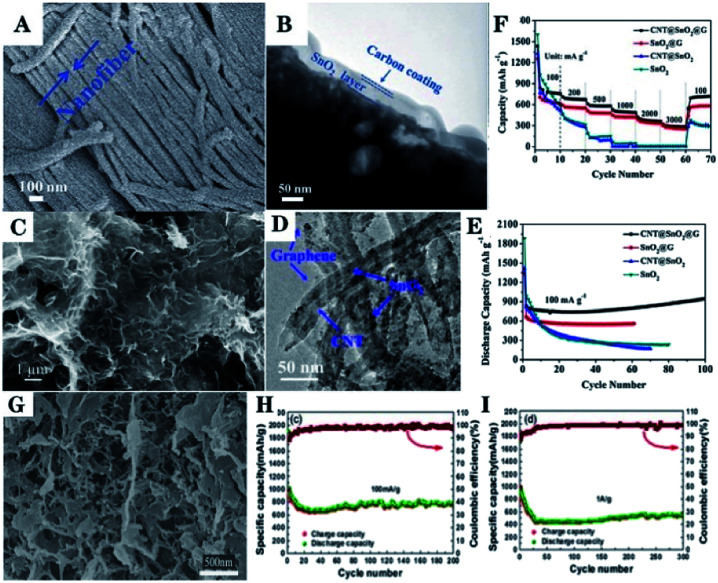
(A) FESEM image, and (B) TEM image of C@SnO_2_@C nanofiber bundle (adapted with permission from ref. 162 copyright 2016 Royal Society of Chemistry). (C) FESEM image, (D) TEM image, (E) cycling performance, and (F) rate performance of CNT@SnO_2_@G (adapted with permission from ref. 263 copyright 2017 Elsevier). (G) SEM image, (H) cycling performance, and (I) rate performance of SnO_2_@G-SWCNT materials (adapted with permission from ref. 289 copyright 2017 American Chemical Society).

A sandwich structure with carbon nanofiber, SnO_2_, and nanofiber bundle for carbon-coating (C@SnO_2_@C) has been fabricated by using collagen fiber (CF), which is a typical fibrous protein extracted from cattle skin and is used as a bio-template as well as the carbon source. FESEM image (Fig. 10A) and TEM image (Fig. 10B) show that the average diameter of the SnO_2_ nanofiber bundle was about 5–10 μm and a layer of SnO_2_ was sandwiched between the carbon nanofiber and the carbon coating layer. Such hierarchical architectures of the C@SnO_2_@C nanofiber bundle guaranteed a good balance between electron transport and Li^+^ diffusion kinetics. Thus, efficient ambipolar diffusion and reduced volume changes of SnO_2_ were obtained to ensure structural integrity with high cycling stability.^162^

A porous 3D core–shell structured CNT@SnO_2_ composite, with a graphene coating (CNT@SnO_2_@G), has been synthesized *via* a two-step hydrothermal method. The first step consists of the synthesis of CNT@SnO_2_, and then, CNT@SnO_2_@G is formed in the subsequent step. FESEM image (Fig. 10C) and TEM image (Fig. 10D) show that CNT@SnO_2_ (about 20–40 nm in width) particles was distributed across the graphene sheets and was encased in the graphene coating, which suppressed the formation of SEI layers on the surface of the CNT@SnO_2_@G. The as-prepared CNT@SnO_2_@G electrode exhibited outstanding lithium storage capability, including a large specific capacity, remarkable cycling stability, and excellent rate capability (Fig. 10E and F).^265^

SnO_2_ nanoparticles anchored on an aerogel based on 3D graphene-single walled carbon nanotube (SnO_2_@G-SWCNT), as shown in Fig. 10G, has been fabricated by a hydrothermal self-assembly process.^291^ The 3D G-SWCNT matrix provides a flexible conductive matrix and a more porous network to support SnO_2_. This is beneficial for facilitating electronic and ionic transportation and mitigating the volume changes of the SnO_2_ during lithiation/delithiation; thus, leading to enhanced electrochemical performance of the SnO_2_ anodes for LIBs. The discharge capacity remained 758 mA h g^−1^ at 100 mA g^−1^ after 200 cycles (Fig. 10H) and 537 mA h g^−1^ at 1 A g^−1^ after 300 cycles (Fig. 10I).^291^

## 4. SnO_2_/TMOs/C

The composites of SnO_2_ and transition metal oxides (TMOs) have been considered as promising anode materials for LIBs.^292–294^ By the introduction of metal oxides, the reversible decomposition of Li_2_O can be increased because TMOs can convert extra Li_2_O into Li^+^;^295–297^ thus, enhancing their cycling stability, rate performance, and rate capability. However, the electrochemical performance of SnO_2_/TMOs is unsatisfactory because of low conductivity and large volume expansion, leading to poor cycling stability.^136^ In order to overcome these problems, much work has been focused on the fabrication of SnO_2_/TMOs/C materials in recent years. Supported and coated by carbonaceous material and TMOs, the structural stability and conductivity of SnO_2_ can be significantly improved. This section will cover synthesis methods, morphology, and electrochemical performance of SnO_2_/TMOs/C.^298–302^

### 4.1 SnO_2_/Fe_2_O_3_/C

Fe_2_O_3_ has been considered as another promising anode material for LIBs due to its large theoretical capacity (1007 mA h g^−1^) and low cost.^303,304^ However, the short carrier diffusion length inhibits lithiation/delithiation processes. It has been reported that SnO_2_/Fe_2_O_3_ showed an improvement in photocatalysis, energy storage, and gas sensing.^303,305–307^ Furthermore, the SnO_2_/Fe_2_O_3_/C electrode showed better lithium storage capability.^308^ In this composite, SnO_2_ possesses a high intrinsic conductivity and a shortened charge diffusion distance, while Fe_2_O_3_ facilitates the reversible decomposition of Li_2_O and prevents Sn aggregation during charging and discharging.^309^

**Fig. 11:**
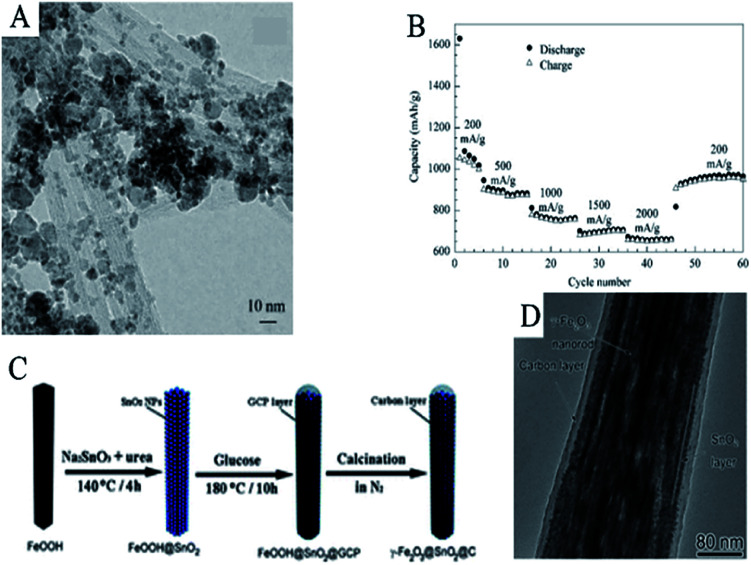
(A) TEM image, and (B) rate performance of SnO_2_/Fe_2_O_3_/SWCNTs (adapted with permission from ref. 308 copyright 2014 Elsevier). (C) TEM images, and (D) schematic illustration of the synthesis of γ-Fe_2_O_3_@SnO_2_@C core–shell nanorods (adapted with permission from ref. 115 copyright 2013 Royal Society of Chemistry).

In 2014, Wu *et al.* proposed a facile hydrothermal method to synthesize a ternary phased SnO_2_/Fe_2_O_3_/SWCNTs composite. As shown in Fig. 11A, the composites of SnO_2_ and Fe_2_O_3_ nanoparticles were well distributed and firmly anchored on to SWCNTs, which serve as a buffer and conductive matrix. Nanosized Fe_2_O_3_/SnO_2_ composites can suppress the effect of volume changes and particle agglomeration. The SnO_2_/Fe_2_O_3_/SWCNTs electrode showed a high reversible capacity, superior cycle performance, and high rate capability. It delivered a capacity of 692 mA h g^−1^ at 200 mA g^−1^ after 50 cycles. Even at a rate as high as 2000 mA g^−1^, this composite could still maintain its capacity at 656 mA h g^−1^ (Fig. 11B).^310^

The growth of SnO_2_ on Fe_2_O_3_ and subsequent carbon coating on SnO_2_/Fe_2_O_3_ is another common synthesis route for SnO_2_/Fe_2_O_3_/C. Du *et al.* synthesized γ-Fe_2_O_3_@SnO_2_@C porous core–shell nanorods. They first formed FeOOH nanorods *via* a hydrothermal process, which served as a template for the subsequent SnO_2_ deposition process in another hydrothermal system. After the deposition of SnO_2_ on the FeOOH nanorods, the as-synthesized FeOOH@SnO_2_ nanorods were coated with a carbon layer *via* another hydrothermal process and carbonized under N_2_ at 500 °C for 2 h (Fig. 11C). The TEM image (Fig. 11D) shows that SnO_2_ can successfully grow on the Fe_2_O_3_ nanostructure with well-defined interfaces. The thickness of the SnO_2_ layer is 5–10 nm, which is composed of SnO_2_ nanoparticles with a diameter of about 3–5 nm, and the core nanorod is highly porous. Such a porous core–shell hybrid nanorod-based electrode showed good cycling and rate performance due to the improvement of conductivity and structural stability by the introduction of carbon and Fe_2_O_3_.^115^

### 4.2 SnO_2_/Co_3_O_4_/C

**Fig. 12:**
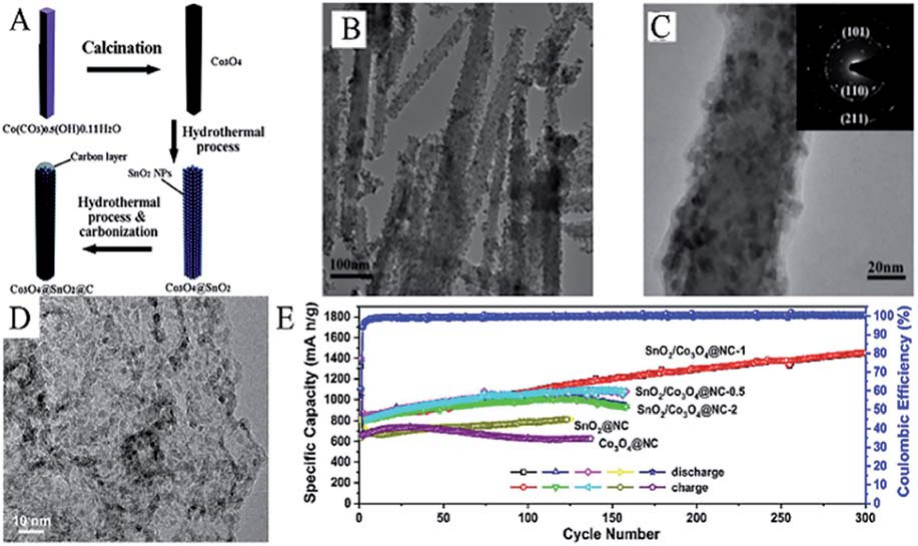
(A) Schematic illustration of the fabrication of Co_3_O_4_@SnO_2_@C core–shell nanorods. (B) TEM image, and (C) HRTEM images of Co_3_O_4_@SnO_2_@C core–shell nanorods (adapted with permission from ref. 313 copyright 2012 Royal Society of Chemistry). (D) HRTEM image and, (E) cycling performance of SnO_2_/Co_3_O_4_@NC-1 nanoflakes (adapted with permission from ref. 119 copyright 2019 Elsevier).

Many studies have concentrated on improving electrochemical properties of SnO_2_ and Co_3_O_4_ by designing and synthesizing unique nanostructures. Like most transition metals, the presence of Co can improve the reversibility of the reduction reaction of Li_2_O and further enhance the reversible capacity.^311–315^ As shown in Fig. 12A, Co_3_O_4_@SnO_2_@C core–shell nanorods have been synthesized *via* a hydrothermal method followed by carbonization. Fig. 12B and C show that SnO_2_ nanoparticles uniformly coated the surface of Co_3_O_4_ nanorods and that the coating was smooth and uniform, with a thickness of 5–10 nm. Such materials exhibited improved cycling performance and higher specific capability as an anode material for LIBs (860 mA h g^−1^ after 50 cycles at 0.2 A g^−1^). This result demonstrated that the combination of SnO_2_ and Co_3_O_4_, into an integrated core–shell nanorod structure, exhibited a better and more elegant synergistic effect during the charge/discharge processes.

SnO_2_/Co_3_O_4_@N-doped carbon (NC) usually shows better lithium storage properties compared to most SnO_2_/Co_3_O_4_/C materials.^316^ Wang *et al.* successfully designed and fabricated SnO_2_/Co_3_O_4_/NC nanoflakes *via* a combined strategy of CVD and template synthesis.^119,317^ HRTEM images (Fig. 12D) show that SnO_2_/Co_3_O_4_ nanoparticles were uniformly distributed on the NC nanoflakes. By adjusting the ratio of the precursor and reaction conditions, they found that the SnO_2_/Co_3_O_4_@NC (*R*_Sn/Co_ = 1 : 1) nanoflakes-based electrode demonstrated excellent lithium storage capability, *i.e.*, a discharge capacity of 1450.3 mA h g^−1^ after 300 cycles at 200 mA h g^−1^ (Fig. 12E). The superior lithium storage of such materials may result from the synergistic effect between the combination of SnO_2_ and Co_3_O_4_ and better conductivity caused by the N-doped carbon matrix.^119^

Therefore, in SnO_2_/Co_3_O_4_/C composites, SnO_2_ and Co_3_O_4_ display a synergistic enhancement effect. It can provide additional active sites for lithium storage and shorten the lithium diffusion distance. Additionally, the carbon layer can greatly improve the electrode conductivity and restrain volume changes during the lithiation/delithiation processes.

### 4.3 SnO_2_/TiO_2_/C

SnO_2_, as one of the most extensively investigated anode material, which offers a high specific capacity. However, poor cycling stability, due to large volume expansion and subsequent pulverization during Li^+^ insertion/extraction processes, greatly restricts its application as an anode material.^130,318–320^ In contrast, TiO_2_ exhibits negligible volume change (less than 4%) and stable electrochemical properties, but its application is limited due to its low specific capacity.^321–325^ Furthermore, SnO_2_ and TiO_2_ have complementary characteristics in LIBs, *i.e.*, Sn^4+^ and Ti^4+^ possess similar radii and the lattice matching of SnO_2_ and TiO_2_ is good.^326–328^ Considering the facts mentioned above, in order to promote cycling stability of SnO_2_, various SnO_2_/TiO_2_ based hybrid materials have been synthesized.^329–334^

**Fig. 13:**
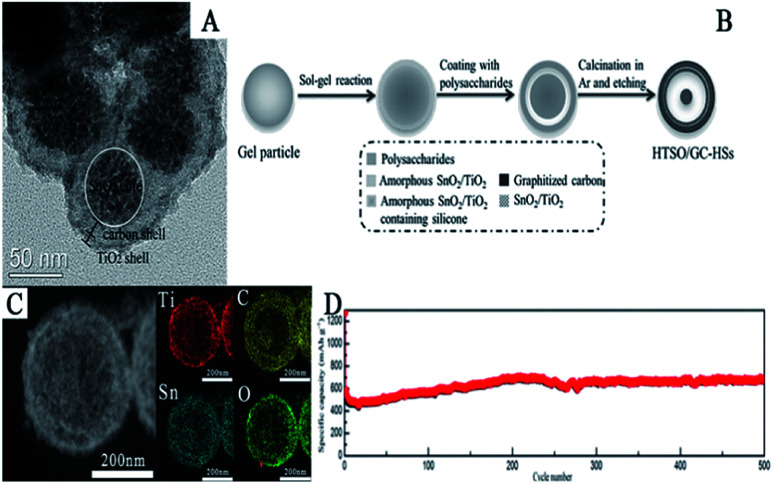
(A) TEM images of SnO_2_@C@TiO_2_ nanochains (adapted with permission from ref. 296 copyright 2015 Elsevier). (B) Schematic illustration of the synthesis, (C) STEM image, and (D) cycling performance of HTSO/GC-HSs (adapted with permission from ref. 299 copyright 2017 Wiley).

Mesoporous SnO_2_@C@TiO_2_ nanochains have been synthesized by first fabricating SnO_2_@C core–shell nanochains *via* a hydrothermal method and subsequent continuous mechanical stirring of the solution of SnO_2_@C and tetrabutyl titanate (C_16_H_36_O_4_Ti). It is noticeable that the SnO_2_ core is composed of SnO_2_ nanoparticles with a diameter of 2–6 nm and is coated by a thin carbon layer (2–6 nm) as well as a TiO_2_ layer (about 8 nm) (Fig. 13A). In this 3D hierarchical structure, the carbon layer and the TiO_2_ layer can effectively improve cycling stability and discharge capacity, *i.e.*, the initial discharge capacity of 807 mA h g^−1^ and 369 mA h g^−1^ after 100 cycles at 100 mA h g^−1^.^298^

Xie *et al.* reported the synthesis of hierarchical TiO_2_/SnO_2_ hollow spheres coated with graphitized carbon (HTSO/GC-HSs) by a multi-step approach. As shown in Fig. 13B, titanate-silicone gel particles were first reacted with SnCl_2_*via* a sol–gel process to obtain core–shell hybrid structures, which were further coated with a polysaccharide *via* a hydrothermal process and subsequent carbonization in Ar atmosphere. As shown in Fig. 13C, the as-prepared mesoporous HTSO/GC-HSs had a yolk–shell structure and elements Sn, Ti, O and C were found to be uniformly distributed in this hierarchical hollow nanostructure. Additionally, they also found that due to the uniform distribution of SnO_2_, TiO_2_, and the carbon layer, a solid solution was formed which could effectively suppress the effect of volume changes during the charge/discharge process. The specific discharge capacity remained at about 680 mA h g^−1^ at 1 A g^−1^ after 500 cycles, which demonstrates the excellent cycling performance of HTSO/GC-HSs at high current densities (Fig. 13D).^301^

SnO_2_/TiO_2_/C combines the high capacity of SnO_2_ with the long cycle life and high rate capability of TiO_2_.^335,336^ Furthermore, carbonaceous materials can inhibit agglomeration and pulverization of SnO_2_ and enhance the conductivity of TiO_2_.^337,338^ Therefore, SnO_2_/TiO_2_/C present an important area of research for the future.

### 4.4 SnO_2_/TMOs/graphene

In the previous sections, we have discussed the synthesis methods, morphologies, and electrochemical performance of some representative SnO_2_/TMOs/C anode materials. We classified them into different categories according to metal oxides because different TMOs greatly influence the physical and chemical properties of such complex materials due to different reaction mechanisms. In addition, different carbonaceous materials used in SnO_2_/TMOs/C also influence the electrode performance in LIBs. Among these complex materials, much work has been focused on the fabrication of SnO_2_/TMOs/graphene materials in recent years. Compared to amorphous carbon and CNTs, graphene shows higher lithium storage capacity.^339–342^ Additionally, graphene, as a supportive matrix and conductive network, displays excellent mechanical properties and electronic conductivity, which is conducive to improvement in cycling stability and rate performance of SnO_2_. Therefore, effectively combining SnO_2_, TMOs, and graphene, to synthesize high-performance and practical anode material, has been a popular research subject in recent years.^46,275,343–345^

**Fig. 14:**
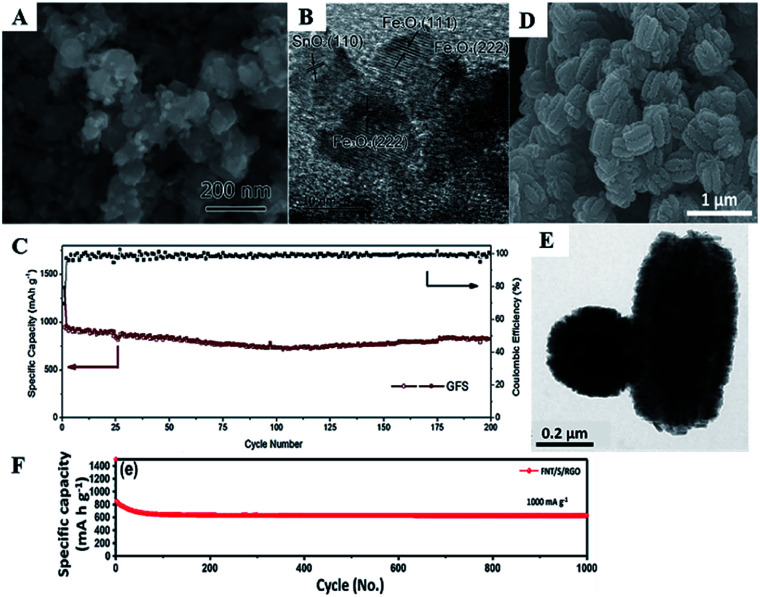
(A) SEM image, (B) HRTEM image, and (C) cycling performance of FSG (adapted with permission from ref. 132 copyright 2016 Elsevier). (D) SEM image, (E) TEM image from top and side view of CNT/S, and (F) cycling performance of FNT/S/RGO (adapted with permission from ref. 136 copyright 2017 Elsevier).

Wang *et al.* synthesized a Fe_3_O_4_/SnO_2_/rGO (FSG) composite *via* a facile hydrothermal method. SEM images (Fig. 14A) and elemental mapping show that FSG consists of SnO_2_ and Fe_3_O_4_ nanoparticles with diameters of about 10–100 nm, uniformly distributed on the surface of the rGO. In this composite, rGO served as a conductive and robust matrix to prevent aggregation of Fe_3_O_4_ and SnO_2_ nanoparticles on rGO. As shown in Fig. 14B, the nanocrystallites of Fe_3_O_4_ and SnO_2_ tend to link with each other, which is beneficial for suppressing both, *i.e.*, the formation of the SEI film and the volume changes. Such a novel nanostructure can effectively shorten the transport path of Li^+^ and electrons and helps improve electrolyte penetration. The FSG nanocomposite exhibited a reversible capacity of 947 mA h g^−1^ at a current density of 200 mA g^−1^ in the first cycle and maintained a capacity of 831 mA h g^−1^ after 200 cycles (Fig. 14C).^132^

Lee *et al.* have synthesized hollow nanostructured α-Fe_2_O_3_ nanotubes/SnO_2_/rGO (FNT/S/G) *via* a microwave-assisted hydrothermal method. As shown in Fig. 14D, SnO_2_ nanorods grow on the surface of FNT, and FNT/SnO_2_ are uniformly anchored on rGO sheets. The diameter and length (Fig. 14E) of FNT/S are about 230 nm and 660 nm, respectively. Lee *et al.* also compared the electrochemical performance of FNT, FNT/S, and FNT/S/G. As shown in Fig. 14F, FNT/S/G exhibited the highest discharge capacity under all tested current densities. FNT/S was the second best, demonstrating that the introduction of SnO_2_ and rGO can effectively enhance the lithium storage capability and cycling stability of the anode material. The specific discharge capacity of FNT/S/RGO remained at 629 mA h g^−1^ at 1 A g^−1^ after 1000 cycles.^136,346^

Other SnO_2_/TMOs/graphene composites like SnO_2_/TiO_2_/GN,^338^ SnO_2_/CuO/GN^347^ and SnO_2_/In_2_O_3_/GN^348^ also exhibit improved electrochemical performance. The introduction of TMOs and graphene effectively improve the lithium storage capacity of SnO_2_. Graphene serves as a supportive and conductive matrix that inhibits agglomeration and pulverization of SnO_2_ and TMOs during the charge/discharge process.

## 5. Other SnO_2_-based compounds

**Fig. 15:**
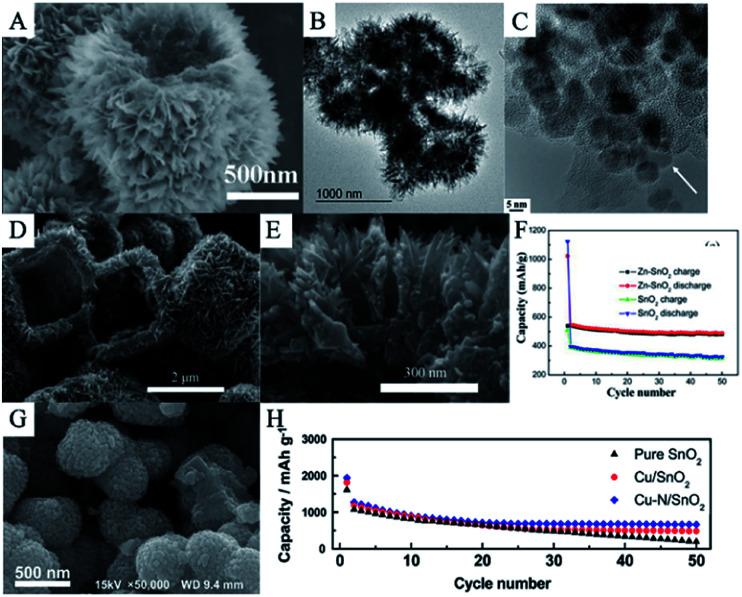
5(A) SEM image, and (B) TEM image of LTO–SnO_2_ composites (adapted with permission from ref. 142 copyright 2018 Elsevier). (C) HRTEM image of carbon-coated Fe-doped SnO_2_ nanoparticles (adapted with permission from ref. 351 copyright 2015 Elsevier). (D and E) SEM images, and (F) cycling performance of Zn-doped SnO_2_ nanomaterials (adapted with permission from ref. 355 copyright 2014 Elsevier). (G) SEM image, and (H) cycling performance of Cu/N-doped SnO_2_ (adapted with permission from ref. 139 copyright 2014 Elsevier).

Li_4_Ti_5_O_12_ (LTO) has been considered as a promising anode material for LIBs due to its excellent cycling stability during lithiation/delithiation.^349,350^ However, like TiO_2_, LTO possesses a lower theoretical capacity than other anode materials (175 mA h g^−1^) and shows poor rate performance.^351,352^ Therefore, in order to improve lithium storage of LTO and cycling performance of SnO_2_, an effective strategy is to synthesize composites of SnO_2_ and LTO nanostructures. Ding *et al.* synthesized hierarchical yolk–shell LTO–SnO_2_ structures *via* a two-step hydrothermal method. They first synthesized SnO_2_ microspheres at 180 °C for 12 h in a hydrothermal system and then, coated LTO on the surface of the SnO_2_ in another hydrothermal system, followed by calcination at 600 °C for 3 h. SEM images (Fig. 15A) and TEM images (Fig. 15B) clearly show that the yolks are SnO_2_ microspheres composed of SnO_2_ nanoparticles, while the shells consist of LTO nanosheets. Hence, this hierarchical coating structure can effectively improve structural stability and suppress the volume changes of SnO_2_ microspheres; thus, improving the cycling performance and showing high rate capacity.^142^

Heteroatom-doped SnO_2_ reveals better electrochemical performance compared to pure SnO_2_. The presence of a dopant may favor the reversible formation of Li_2_O and improve the conductivity of SnO_2_; thus, enhancing the specific capacity of the anode material.^353^ Various heteroatom-doped SnO_2_ nanomaterials have been synthesized and tested as anode materials in LIBs.^354–356^ For example, Mueller *et al.* have synthesized carbon-coated Fe-doped SnO_2_ nanoparticles (Fig. 15C). The discharge capacity of such materials is about 1726 mA h g^−1^ after 10 cycles at 50 mA g^−1^, which is around twice the theoretical capacity of pure SnO_2_.^353^ Zn-doped SnO_2_ hierarchical cube-like nanomaterials were fabricated *via* hydrothermal method. As shown in Fig. 15D and E, the cube is about 2 μm in length and composed of assembled nanorods which are 20–40 nm in diameter. The discharge capacity of Zn-doped SnO_2_ composites is 488.3 mA h g^−1^ after 50 cycles at 10 mA g^−1^ (Fig. 15F).^357^ Wan *et al.* fabricated Cu/N-doped SnO_2_ nanocomposites, as shown in Fig. 15G. They found that with the introduction of Cu/N, the average diameter of SnO_2_ became smaller, and the surface became rough, which is beneficial for ion diffusion and suppressing volume expansion. Cu/N-doped SnO_2_ electrode materials are known to deliver a discharge capacity of 1939 mA h g^−1^ in the first cycle and remain at 664 mA h g^−1^ after 50 cycles at 0.1C.^139^

## 6. Conclusions

In this review, we summarized various SnO_2_-based nanomaterials as anode materials for lithium-ion batteries (Table 1). By optimizing the structure and composition of the materials, *i.e.*, by synthesizing nanostructured SnO_2_ and making composites with carbonaceous materials or transition metal oxides (TMOs), the surface area and reaction sites of the anode materials can be effectively increased, improving the electrochemical properties of SnO_2_-based anode materials. The introduction of carbonaceous materials or TMOs can effectively buffer the internal stresses caused by large volume changes, reduce the irreversible capacity loss, and improve the cycle performance. These aspects lay the foundation for the development and commercialization of high-performance LIBs in the future. Since lithium storage in SnO_2_ is accompanied by repeatedly inserting and removing lithium ions, volume changes and irreversible capacity loss of SnO_2_ electrode cannot be completely avoided. Significant research on nanosized SnO_2_-based anode materials has been carried out, and while these materials display superior lithium storage capacity and cycling stability, critical issues like volume expansion and pulverization of SnO_2_ remain to be solved. Further extensive research is needed before new, non-carbonaceous anode materials, such as SnO_2_-based nanomaterials, can be commercialized.

**Table 1:** Electrochemical property comparison table of some typical SnO_2_-based anode materials for LIBs

Materials	Morphology		Preparation approach	Voltage window (V)	Current density (A g^−1^)	Cycle number	Specific capacity (mA h g^−1^)	Reference
SnO_2_ nanorods	Diameter of 60 nm and length of 670 nm	Hydrothermal		0.005–2.5	0.078	100	580	154
SnO_2_ nanotube arrays	Diameter of 100–300 nm and thickness of 10–20 nm	Solvothermal and annealing		0.005–2	0.078	20	750–800	64
SnO_2_ nanowires	Diameter of 6 nm and length of >3 μm	Solvothermal and annealing		0–1.2	0.156	50	773	71
SnO_2_ nanosheet arrays	Thickness of 20 nm and length of 500 nm	Hydrothermal		0.01–1.2	0.391	50	674.9	79
Hollow SnO_2_ nanospheres	Size of 100–250 nm	Solvothermal and hydrothermal		0.01–2	0.391	40	1140	86
Urchin-like SnO_2_ nanospheres	Diameter of 300 nm	Solvothermal		0.01–3	0.1	50	719	210
SnO_2_ nanocrystals/MWCNT composites	Crystal size of 5 nm	Solvothermal and heat treatment		0.01–3	0.1	100	402	223
CNT@void@SnO_2_@C	Tube in tube diameter of 100–150 nm	Spray drying		0.01–3	0.2	350	702.5	226
SnO_2_/C composite hollow spheres	Size of 150–400 nm	Hydrothermal and carbonation		0–2.5	0.16	50	473	99
SnO_2_ nanoparticles/carbon composite	Nanoparticles of 5–10 nm	Polyol method		0.005–1.5	0.2	100	370	251
SnO_2_ nanosheets/GO	Length of 100 nm and thickness of 5–10 nm	Hydrothermal		0.01–1.2	0.4	50	518	171
SnO_2_ nanoparticles/graphene	Particle size of 3–8 nm	Microwave-assisted hydrothermal		0.01–3	0.2	100	978.6	269
SnO_2_@rGO-Cl	Nanoparticle size of 5 nm	Heat treatment		0.01–3	0.2	400	1008	275
CNT@SnO_2_@G	Width of 20–40 nm	Hydrothermal		0.01–3	0.1	100	947	265
SnO_2_@G-SWCNT	Diameter of 3–5 nm and particle size of 6–8 nm	Hydrothermal		0.01–3	1	300	537	291
SnO_2_–Fe_2_O_3_/SWCNTs nanocomposite	Particle size of 10–50 nm	Hydrothermal		0.01–3	0.2	50	692	310
Co_3_O_4_@SnO_2_@C core–shell nanorods	Thickness of 5–10 nm	Hydrothermal		0.01–2.5	0.2	50	860	313
SnO_2_@C@TiO_2_ nanochains	SnO_2_ for 2–6 nm; thickness of 2–6 nm and 8 nm for carbon and TiO_2_	Hydrothermal and subsequent mechanical stirring		0.01–3	0.1	100	369	298
Fe_2_O_3_ nanotubes/SnO_2_/rGO	Diameter of 230 nm and length of 660 nm	Microwave-assisted hydrothermal		0.01–3	1	1000	629	136
Yolk–shell LTO–SnO_2_	Diameter of 1.0–1.5 μm	Hydrothermal and calcination		0.01–3	0.175	200	253.2	142
Fe-doped SnO_2_ nanoparticles	Diameter of 15 nm	Hydrothermal and calcination		0.01–2	0.05	10	1726	351

## Conflicts of interest

There are no conflicts to declare.

## Supplementary Material

## References

[cit1] Tarascon J. M., Armand M. (2001). Nature.

[cit2] Nitta N., Wu F., Lee J. T., Yushin G. (2015). Mater. Today.

[cit3] Reddy M. V., Rao G. V. S., Chowdari B. V. R. (2013). Chem. Rev..

[cit4] Etacheri V., Marom R., Elazari R., Salitra G., Aurbach D. (2011). Energy Environ. Sci..

[cit5] Liu C., Li F., Ma L. P., Cheng H. M. (2010). Adv. Mater..

[cit6] Kim M.-S., Fang B., Kim J. H., Yang D., Kim Y. K., Bae T.-S., Yu J.-S. (2011). J. Mater. Chem..

[cit7] Li W., Zhao L., Tian Y., Gong Y., Huang X., Cui Z., Zeng R. (2014). Electrochim. Acta.

[cit8] Cabana J., Monconduit L., Larcher D., Rosa Palacin M. (2010). Adv. Mater..

[cit9] Magasinski A., Dixon P., Hertzberg B., Kvit A., Ayala J., Yushin G. (2010). Nat. Mater..

[cit10] Scrosati B., Hassoun J., Sun Y.-K. (2011). Energy Environ. Sci..

[cit11] Fang B., Kim M.-S., Kim J. H., Lim S., Yu J.-S. (2010). J. Mater. Chem..

[cit12] Goodenough J. B., Kim Y. (2010). Chem. Mater..

[cit13] Jiang J., Li Y., Liu J., Huang X., Yuan C., Lou X. W. (2012). Adv. Mater..

[cit14] Chang K., Chen W. (2011). ACS Nano.

[cit15] Sun Y., Wu Q., Shi G. (2011). Energy Environ. Sci..

[cit16] Wu Z.-S., Zhou G., Yin L.-C., Ren W., Li F., Cheng H.-M. (2012). Nano Energy.

[cit17] Yuan C., Wu H. B., Xie Y., Lou X. W. (2014). Angew. Chem., Int. Ed..

[cit18] Liu N., Lu Z., Zhao J., McDowell M. T., Lee H.-W., Zhao W., Cui Y. (2014). Nat. Nanotechnol..

[cit19] Goodenough J. B., Park K.-S. (2013). J. Am. Chem. Soc..

[cit20] Bruce P. G., Freunberger S. A., Hardwick L. J., Tarascon J.-M. (2012). Nat. Mater..

[cit21] Deng Y., Fang C., Chen G. (2016). J. Power Sources.

[cit22] Lu L., Han X., Li J., Hua J., Ouyang M. (2013). J. Power Sources.

[cit23] Choi J. W., Aurbach D. (2016). Nat. Rev. Mater..

[cit24] Hua W.-B., Guo X.-D., Zheng Z., Wang Y.-J., Zhong B.-H., Fang B., Wang J.-Z., Chou S.-L., Liu H. (2015). J. Power Sources.

[cit25] Xing Y., Wang S., Fang B., Feng Y., Zhang S. (2018). Microporous Mesoporous Mater..

[cit26] Hua W., Wang Y., Zhong Y., Wang G., Zhong B., Fang B., Guo X., Liao S., Wang H. (2015). Chin. J. Chem..

[cit27] Cheng F., Chen Y., Sun A., Zhou X., Yang J., Tang J. (2019). Ceram. Int..

[cit28] Jia J., Hu X., Wen Z. (2018). Nano Res..

[cit29] Wang T., Li H., Shi S., Liu T., Yang G., Chao Y., Yin F. (2017). Small.

[cit30] Xu G., Zhang L., Guo C., Gu L., Wang X., Han P., Zhang K., Zhang C., Cui G. (2012). Electrochim. Acta.

[cit31] Xia H., Lai M., Lu L. (2010). J. Mater. Chem..

[cit32] Reddy A. L. M., Shaijumon M. M., Gowda S. R., Ajayan P. M. (2009). Nano Lett..

[cit33] Zhu J., He J. (2012). ACS Appl. Mater. Interfaces.

[cit34] Xu H., Hu X., Yang H., Sun Y., Hu C., Huang Y. (2015). Adv. Energy Mater..

[cit35] Sun B., Chen Z., Kim H.-S., Ahn H., Wang G. (2011). J. Power Sources.

[cit36] Kong D., Luo J., Wang Y., Ren W., Yu T., Luo Y., Yang Y., Cheng C. (2014). Adv. Funct. Mater..

[cit37] Zhu S., Li J., Deng X., He C., Liu E., He F., Shi C., Zhao N. (2017). Adv. Funct. Mater..

[cit38] Wang H., Cui L.-F., Yang Y., Casalongue H. S., Robinson J. T., Liang Y., Cui Y., Dai H. (2010). J. Am. Chem. Soc..

[cit39] Gao J., Lowe M. A., Abruna H. D. (2011). Chem. Mater..

[cit40] Cai Z., Xu L., Yan M., Han C., He L., Hercule K. M., Niu C., Yuan Z., Xu W., Qu L., Zhao K., Mai L. (2015). Nano Lett..

[cit41] Zhu X., Zhu Y., Murali S., Stollers M. D., Ruoff R. S. (2011). ACS Nano.

[cit42] Zhang L., Wu H. B., Madhavi S., Hng H. H., Lou X. W. (2012). J. Am. Chem. Soc..

[cit43] Xu X., Cao R., Jeong S., Cho J. (2012). Nano Lett..

[cit44] Wang Z., Luan D., Madhavi S., Hu Y., Lou X. W. (2012). Energy Environ. Sci..

[cit45] Reddy M. V., Yu T., Sow C.-H., Shen Z. X., Lim C. T., Rao G. V. S., Chowdari B. V. R. (2007). Adv. Funct. Mater..

[cit46] Zhou G., Wang D.-W., Li F., Zhang L., Li N., Wu Z.-S., Wen L., Lu G. Q., Cheng H.-M. (2010). Chem. Mater..

[cit47] Zhang W.-M., Wu X.-L., Hu J.-S., Guo Y.-G., Wan L.-J. (2008). Adv. Funct. Mater..

[cit48] Wei W., Yang S., Zhou H., Lieberwirth I., Feng X., Muellen K. (2013). Adv. Mater..

[cit49] Luo J., Liu J., Zeng Z., Ng C. F., Ma L., Zhang H., Lin J., Shen Z., Fan H. J. (2013). Nano Lett..

[cit50] He C., Wu S., Zhao N., Shi C., Liu E., Li J. (2013). ACS Nano.

[cit51] Wang X., Wu X.-L., Guo Y.-G., Zhong Y., Cao X., Ma Y., Yao J. (2010). Adv. Funct. Mater..

[cit52] Lou X. W., Deng D., Lee J. Y., Feng J., Archer L. A. (2008). Adv. Mater..

[cit53] Li Y., Tan B., Wu Y. (2008). Nano Lett..

[cit54] Li W. Y., Xu L. N., Chen J. (2005). Adv. Funct. Mater..

[cit55] Zhang W. M., Hu J. S., Guo Y. G., Zheng S. F., Zhong L. S., Song W. G., Wan L. J. (2008). Adv. Mater..

[cit56] Lou X. W., Wang Y., Yuan C. L., Lee J. Y., Archer L. A. (2006). Adv. Mater..

[cit57] Lou X. W., Li C. M., Archer L. A. (2009). Adv. Mater..

[cit58] Guo C., Cao M., Hu C. (2004). Inorg. Chem. Commun..

[cit59] Liu B., Zhang J.-G., Xu W. (2018). Joule.

[cit60] Courtney I. A. (1997). J. Electrochem. Soc..

[cit61] Zhou X., Wan L.-J., Guo Y.-G. (2013). Adv. Mater..

[cit62] Kim H., Park G. O., Kim Y., Muhammad S., Yoo J., Balasubramanian M., Cho Y.-H., Kim M.-G., Lee B., Kang K., Kim H., Kim J. M., Yoon W.-S. (2014). Chem. Mater..

[cit63] Park M.-S., Wang G.-X., Kang Y.-M., Wexler D., Dou S.-X., Liu H.-K. (2007). Angew. Chem., Int. Ed..

[cit64] Wang J., Du N., Zhang H., Yu J., Yang D. (2011). J. Phys. Chem. C.

[cit65] Wang Y., Wu M., Jiao Z., Lee J. Y. (2009). Nanotechnology.

[cit66] Lai M., Gonzalez Martinez J. A., Grätzel M., Riley D. J. (2006). J. Mater. Chem..

[cit67] Du N., Zhang H., Chen B., Ma X., Yang D. (2008). Chem. Commun..

[cit68] Wang Y., Lee J. Y., Zeng H. C. (2005). Chem. Mater..

[cit69] Ding S. J., Wang Z. Y., Madhavi S., Lou X. W. (2011). J. Mater. Chem..

[cit70] Han Y. T., Wu X., Ma Y. L., Gong L. H., Qu F. Y., Fan H. J. (2011). CrystEngComm.

[cit71] Kim H., Cho J. (2008). J. Mater. Chem..

[cit72] Ko Y. D., Kang J. G., Park J. G., Lee S., Kim D. W. (2009). Nanotechnology.

[cit73] Ren W., Wang C., Lu L., Li D., Cheng C., Liu J. (2013). J. Mater. Chem. A.

[cit74] Wan Q., Dattoli E. N., Lu W. (2007). Appl. Phys. Lett..

[cit75] Tian Q., Li L., Chen J., Yang L., Hirano S.-i. (2018). J. Power Sources.

[cit76] Zhu X., Shi H., Yin J., Zhu H., Zhou Y., Tang Y., Wu P., Lu T. (2014). RSC Adv..

[cit77] Zhang L., Wu H. B., Wen Lou X. (2014). Mater. Horiz..

[cit78] Zhang S., Yin B., Jiao Y., Liu Y., Qu F., Wu X. (2014). Appl. Surf. Sci..

[cit79] Zhao X., Liu B., Hu C., Cao M. (2014). Chemistry.

[cit80] Zhu Y., Guo H., Zhai H., Cao C. (2015). ACS Appl. Mater. Interfaces.

[cit81] Wei W., Du P., Liu D., Wang H., Liu P. (2017). J. Colloid Interface Sci..

[cit82] Kumar B., Lee D. H., Kim S. H., Yang B., Maeng S., Kim S. W. (2010). J. Phys. Chem. C.

[cit83] Park G. D., Lee J.-K., Kang Y. C. (2017). Adv. Funct. Mater..

[cit84] Kim J. H., Jeon K. M., Park J.-S., Kang Y. C. (2017). J. Power Sources.

[cit85] Park J.-S., Oh Y. J., Kim J. H., Kang Y. C. (2020). Mater. Charact..

[cit86] Lou X. W., Wang Y., Yuan C., Lee J. Y., Archer L. A. (2006). Adv. Mater..

[cit87] Ding S., Wen Lou X. (2011). Nanoscale.

[cit88] Wang H., Fu F., Zhang F., Wang H.-E., Kershaw S. V., Xu J., Sun S.-G., Rogach A. L. (2012). J. Mater. Chem..

[cit89] Wang Y., Su T., Chen H., Liu W., Dong Y., Hu S. (2014). Mater. Lett..

[cit90] Qin J., Zhao N., Shi C., Liu E., He F., Ma L., Li Q., Li J., He C. (2017). J. Mater. Chem. A.

[cit91] Kim W.-S., Hwa Y., Jeun J.-H., Sohn H.-J., Hong S.-H. (2013). J. Power Sources.

[cit92] Zhang J., Ren H., Wang J., Qi J., Yu R., Wang D., Liu Y. (2016). J. Mater. Chem. A.

[cit93] Jiang L.-Y., Wu X.-L., Guo Y.-G., Wan L.-J. (2009). J. Phys. Chem. C.

[cit94] Zhang H.-X., Feng C., Zhai Y.-C., Jiang K.-L., Li Q.-Q., Fan S.-S. (2009). Adv. Mater..

[cit95] Dong W., Xu J., Wang C., Lu Y., Liu X., Wang X., Yuan X., Wang Z., Lin T., Sui M., Chen I. W., Huang F. (2017). Adv. Mater..

[cit96] Zhang C., Peng X., Guo Z., Cai C., Chen Z., Wexler D., Li S., Liu H. (2012). Carbon.

[cit97] Long W., Fang B., Ignaszak A., Wu Z., Wang Y.-J., Wilkinson D. (2017). Chem. Soc. Rev..

[cit98] Noerochim L., Wang J.-Z., Chou S.-L., Li H.-J., Liu H.-K. (2010). Electrochim. Acta.

[cit99] Lou X. W., Deng D., Lee J. Y., Archer L. A. (2008). Chem. Mater..

[cit100] Wen Z., Wang Q., Zhang Q., Li J. (2007). Adv. Funct. Mater..

[cit101] Guan D., Li J., Gao X., Yuan C. (2015). RSC Adv..

[cit102] Zhou X., Liu W., Yu X., Liu Y., Fang Y., Klankowski S., Yang Y., Brown J. E., Li J. (2014). ACS Appl. Mater. Interfaces.

[cit103] Chen J. S., Cheah Y. L., Chen Y. T., Jayaprakash N., Madhavi S., Yang Y. H., Lou X. W. (2009). J. Phys. Chem. C.

[cit104] Zhao X., Zhang Z., Yang F., Fu Y., Lai Y., Li J. (2015). RSC Adv..

[cit105] Cheng J., Xin H., Zheng H., Wang B. (2013). J. Power Sources.

[cit106] Song H., Li N., Cui H., Wang C. (2013). J. Mater. Chem. A.

[cit107] Liu H., Huang J., Li X., Liu J., Zhang Y., Du K. (2012). Appl. Surf. Sci..

[cit108] Fan L., Li X., Yan B., Li X., Xiong D., Li D., Xu H., Zhang X., Sun X. (2016). Appl. Energy.

[cit109] Xu H., Shi L., Wang Z., Liu J., Zhu J., Zhao Y., Zhang M., Yuan S. (2015). ACS Appl. Mater. Interfaces.

[cit110] Liu J., Huang J., Hao L., Liu H., Li X. (2013). Ceram. Int..

[cit111] Xu H., Chen J., Wang D., Sun Z., Zhang P., Zhang Y., Guo X. (2017). Carbon.

[cit112] Fang B., Kim J. H., Kim M.-S., Yu J.-S. (2013). Acc. Chem. Res..

[cit113] Xing Y., Wang Y., Zhou C., Zhang S., Fang B. (2014). ACS Appl. Mater. Interfaces.

[cit114] Lu J., Qi D., Deng C., Zhang X., Yang P. (2010). Nanoscale.

[cit115] Du N., Chen Y., Zhai C., Zhang H., Yang D. (2013). Nanoscale.

[cit116] Li F., Luo G., Yu J., Huang W., Xu D., Chen W., Huang X., Yang S., Fang Y., Yu X. (2019). J. Alloys Compd..

[cit117] Wu X., Wu W., Zhou Y., Huang X., Chen W., Wang Q. (2015). Powder Technol..

[cit118] Huang M., Zhao X. L., Li F., Li W., Zhang B., Zhang Y. X. (2015). J. Mater. Chem. A.

[cit119] Wang J., Wang H., Yao T., Liu T., Tian Y., Li C., Li F., Meng L., Cheng Y. (2020). J. Colloid Interface Sci..

[cit120] Deng D., Kim M. G., Lee J. Y., Cho J. (2009). Energy Environ. Sci..

[cit121] Yang Z., Du G., Guo Z., Yu X., Chen Z., Guo T., Zeng R. (2011). Nanoscale.

[cit122] Ansari A., Nematollahi D. (2020). Appl. Catal., B.

[cit123] Zhao B., Mattelaer F., Kint J., Werbrouck A., Henderick L., Minjauw M., Dendooven J., Detavernier C. (2019). Electrochim. Acta.

[cit124] Si L. L., Yuan Z. Q., Liang J. W., Hu L., Zhu Y. C., Qian Y. T. (2014). J. Mater. Chem. A.

[cit125] Xue L.-J., Xu Y.-F., Huang L., Ke F.-S., He Y., Wang Y.-X., Wei G.-Z., Li J.-T., Sun S.-G. (2011). Electrochim. Acta.

[cit126] El-Shinawi H., Schulze A. S., Neumeier M., Leichtweiß T., Janek J. (2014). J. Phys. Chem. C.

[cit127] Tian Q., Chen Y., Zhang F., Zhang W., Sui Z., Yang L. (2020). Appl. Surf. Sci..

[cit128] Jayalakshmi M., Rao M. M., Venugopal N., Kim K.-B. (2007). J. Power Sources.

[cit129] Guo Q., Sun Z., Gao M., Tan Z., Zhang B., Su D. S. (2013). J. Energy Chem..

[cit130] Wei D., Zhong S., Zhang H., Zhang X., Zhu C., Duan J., Li L., Chen Z., Liu P., Zhang G., Duan H. (2018). Electrochim. Acta.

[cit131] Wen G., Liu H., Liang T., Ouyang Y., Tan L., Hu R., Liu J., Zhang Y., Zhu M. (2020). Electrochim. Acta.

[cit132] Wang Y., Zhang H., Hu R., Liu J., van Ree T., Wang H., Yang L., Zhu M. (2017). J. Alloys Compd..

[cit133] Guo W., Wang Y., Zhang F., Rao S., Mao P., Wang D. (2020). Energy Fuels.

[cit134] Si L., Yuan Z., Liang J., Hu L., Zhu Y., Qian Y. (2014). J. Mater. Chem. A.

[cit135] Cao D., Wang H., Li B., Li C., Xie S., Rogach A. L., Niu C. (2016). Electrochim. Acta.

[cit136] Lee K., Shin S., Degen T., Lee W., Yoon Y. S. (2017). Nano Energy.

[cit137] Liu X., Liu F., Sun Q., Ng A. M., Djurisic A. B., Xie M., Liao C., Shih K., Deng Z. (2014). ACS Appl. Mater. Interfaces.

[cit138] Oh H.-S., Nong H. N., Strasser P. (2015). Adv. Funct. Mater..

[cit139] Wan N., Yu P., Sun S., Wu Q., Li T., Bai Y. (2014). Mater. Lett..

[cit140] Liu X., Zhou G., Or S. W., Sun Y. (2014). RSC Adv..

[cit141] Vignesh A., Siddarth A. S., Gokul O. S., Babu B. R. (2014). Int. J. Environ. Sci. Technol..

[cit142] Ding M., Liu H., Zhu J., Zhao X., Pang L., Qin Y., Deng L. (2018). Appl. Surf. Sci..

[cit143] Vo V., Nguyen Thi X. D., Jin Y.-S., Ly Thi G., Nguyen T. T., Duong T. Q., Kim S.-J. (2017). Chem. Phys. Lett..

[cit144] Liu J., Li Y., Huang X., Ding R., Hu Y., Jiang J., Liao L. (2009). J. Mater. Chem..

[cit145] Chen Y. J., Xue X. Y., Wang Y. G., Wang T. H. (2005). Appl. Phys. Lett..

[cit146] Xu W., Zhao K., Niu C., Zhang L., Cai Z., Han C., He L., Shen T., Yan M., Qu L., Mai L. (2014). Nano Energy.

[cit147] Ye J., Zhang H., Yang R., Li X., Qi L. (2010). Small.

[cit148] Zhang D. F., Sun L. D., Yin J. L., Yan C. H. (2003). Adv. Mater..

[cit149] Yang C. , Zhang G., Zhang L. and Ma L., in Advances In Composites, Pts 1 and 2, ed. J. L. Bu, Z. Y. Jiang and S. Jiao, 2011, vol. 150–151, pp. 1387–1390

[cit150] Li C., Wei W., Fang S., Wang H., Zhang Y., Gui Y., Chen R. (2010). J. Power Sources.

[cit151] Guan C., Wang X., Zhang Q., Fan Z., Zhang H., Fan H. J. (2014). Nano Lett..

[cit152] Huang J. Y., Zhong L., Wang C. M., Sullivan J. P., Xu W., Zhang L. Q., Mao S. X., Hudak N. S., Liu X. H., Subramanian A., Fan H., Qi L., Kushima A., Li J. (2010). Science.

[cit153] Thomas R., Mohan Rao G. (2015). J. Mater. Chem. A.

[cit154] Liu J., Li Y., Huang X., Ding R., Hu Y., Jiang J., Liao L. (2009). J. Mater. Chem..

[cit155] Lou X. W., Zeng H. C. (2002). Chem. Mater..

[cit156] Chen J. S., Cheah Y. L., Madhavi S., Lou X. W. (2010). J. Phys. Chem. C.

[cit157] Wang X., Liu W., Yang H., Li X., Li N., Shi R., Zhao H., Yu J. (2011). Acta Mater..

[cit158] Zhang W., Feng L. L., Chen H. Y., Zhang Y. Y. (2019). Nano.

[cit159] Paraguay-Delgado F., Antunez-Flores W., Miki-Yoshida M., Aguilar-Elguezaba A., Santiago P., Diaz R., Ascencio J. A. (2005). Nanotechnology.

[cit160] Ding S., Chen J. S., Lou X. W. (2011). Chem.–Asian J..

[cit161] Du N., Zhang H., Chen B., Ma X., Huang X., Tu J., Yang D. (2009). Mater. Res. Bull..

[cit162] Wang X., Li J., Chen Z., Lei L., Liao X., Huang X., Shi B. (2016). J. Mater. Chem. A.

[cit163] Cheng B., Russell J. M., Shi W. S., Zhang L., Samulski E. T. (2004). J. Am. Chem. Soc..

[cit164] Xi G., Ye J. (2010). Inorg. Chem..

[cit165] Tan L., Wang M. S., Liu Y. J., Xiao X. C., Fan L. Z., Wang Y. D. (2013). Mater. Technol..

[cit166] Wu Z.-S., Ren W., Wen L., Gao L., Zhao J., Chen Z., Zhou G., Li F., Cheng H.-M. (2010). ACS Nano.

[cit167] Zhao Q., Ma L., Zhang Q., Wang C., Xu X. (2015). J. Nanomater..

[cit168] Ye J., Zhang H., Yang R., Li X., Qi L. (2010). Small.

[cit169] Kumar V., Kim J. H., Pendyala C., Chernomordik B., Sunkara M. K. (2008). J. Phys. Chem. C.

[cit170] Han Y., Wu X., Ma Y., Gong L., Qu F., Fan H. (2011). CrystEngComm.

[cit171] Ding S., Luan D., Boey F. Y., Chen J. S., Lou X. W. (2011). Chem. Commun..

[cit172] Wang D., Li X., Wang J., Yang J., Geng D., Li R., Cai M., Sham T.-K., Sun X. (2012). J. Phys. Chem. C.

[cit173] Zhao B., Huang S.-Y., Wang T., Zhang K., Yuen M. M. F., Xu J.-B., Fu X.-Z., Sun R., Wong C.-P. (2015). J. Power Sources.

[cit174] Zhan L., Zhou X., Luo J., Ning X. (2019). Ceram. Int..

[cit175] Hao S., Sun Y., Liu Y., Zhang Y., Hu G. (2016). J. Alloys Compd..

[cit176] Ramasamy S., Nagamony P., Chinnuswamy V. (2018). Mater. Lett..

[cit177] Xing L.-L., Zhao Y.-Y., Zhao J., Nie Y.-X., Deng P., Wang Q., Xue X.-Y. (2014). J. Alloys Compd..

[cit178] Xing G. Z., Wang Y., Wong J. I., Shi Y. M., Huang Z. X., Li S., Yang H. Y. (2014). Appl. Phys. Lett..

[cit179] Fan L. S., Guo Z. K., Zhang Y., Zhang X. Y., Wang M. X., Yin Y. Y., Zhang N. Q., Sun K. N. (2019). Mater. Today Energy.

[cit180] Esquivel D. Y. A., Brown R. K., Knohl S., Schroeder U. (2020). ChemElectroChem.

[cit181] Fu Y., Manthiram A. (2013). Nano Energy.

[cit182] Meng X., Zhang J., Wang Y., Liu H. (2012). Acta Chim. Sin..

[cit183] Jabbour L., Destro M., Gerbaldi C., Chaussy D., Penazzi N., Beneventi D. (2012). J. Mater. Chem..

[cit184] Chang L., Yi Z., Wang Z., Wang L., Cheng Y. (2019). Appl. Surf. Sci..

[cit185] Wang M.-S., Wang Z.-Q., Yang Z.-L., Huang Y., Zheng J., Li X. (2017). Electrochim. Acta.

[cit186] Jia R., Yue J., Xia Q., Xu J., Zhu X., Sun S., Zhai T., Xia H. (2018). Energy Storage Mater..

[cit187] Fan L., Guo Z., Zhang Y., Zhang X., Wang M., Yin Y., Zhang N., Sun K. (2019). Mater. Today Energy.

[cit188] Vayssieres L. (2003). Adv. Mater..

[cit189] Zhan W.-w., Kuang Q., Zhou J.-z., Kong X.-j., Xie Z.-x., Zheng L.-s. (2013). J. Am. Chem. Soc..

[cit190] Liu J., Qiao S. Z., Hartono S. B., Lu G. Q. (2010). Angew. Chem., Int. Ed..

[cit191] Liu J., Yang T., Wang D.-W., Lu G. Q., Zhao D., Qiao S. Z. (2013). Nat. Commun..

[cit192] Yang H. X., Qian J. F., Chen Z. X., Ai X. P., Cao Y. L. (2007). J. Phys. Chem. C.

[cit193] Chang-Chien C.-Y., Hsu C.-H., Lee T.-Y., Liu C.-W., Wu S.-H., Lin H.-P., Tang C.-Y., Lin C.-Y. (2007). Eur. J. Inorg. Chem..

[cit194] Yin Y., Xin S., Wan L., Li C., Guo Y. (2012). Sci. China: Chem..

[cit195] Zhang L., Wu H. B., Liu B., Lou X. W. (2014). Energy Environ. Sci..

[cit196] Liu Y., Dong H., Liu M. L. (2004). Adv. Mater..

[cit197] Wang T., Xu H., Wang Y., Zeng Y., Liu B. (2020). Mater. Lett..

[cit198] Zhou L., Zhang J., Wu Y., Wang W., Ming H., Sun Q., Wang L., Ming J., Alshareef H. N. (2019). Adv. Energy Mater..

[cit199] Chen J. S., Li C. M., Zhou W. W., Yan Q. Y., Archer L. A., Lou X. W. (2009). Nanoscale.

[cit200] Liu S., Xie M., Li Y., Guo X., Ji W., Ding W., Au C. (2010). Sens. Actuators, B.

[cit201] Wu Y.-Z., Brahma S., Weng S.-C., Chang C.-C., Huang J.-L. (2020). J. Alloys Compd..

[cit202] Park G. D., Lee J.-K., Kang Y. C. (2017). Adv. Funct. Mater..

[cit203] Park G. D., Kim J. H., Kang Y. C. (2018). Nanoscale.

[cit204] Cho J. S., Kang Y. C. (2015). Small.

[cit205] Liu L., Xie F., Lyu J., Zhao T., Li T., Choi B. G. (2016). J. Power Sources.

[cit206] Hu H., Wu L., Gebhardt P., Zhang X., Cherevan A., Gerke B., Pöttgen R., Balducci A., Passerini S., Eder D. (2017). CrystEngComm.

[cit207] Tian W., Zhang C., Zhai T., Li S.-L., Wang X., Liao M., Tsukagoshi K., Golberg D., Bando Y. (2013). Chem. Commun..

[cit208] Kang Y., Li Z., Xu K., He X., Wei S., Cao Y. (2019). J. Alloys Compd..

[cit209] Ding S., Chen J. S., Qi G., Duan X., Wang Z., Giannelis E. P., Archer L. A., Lou X. W. (2011). J. Am. Chem. Soc..

[cit210] Deng J., Chen Y., Ma J., Zhang E., Wang T. (2013). J. Nanosci. Nanotechnol..

[cit211] Yin X. M., Li C. C., Zhang M., Hao Q. Y., Liu S., Chen L. B., Wang T. H. (2010). J. Phys. Chem. C.

[cit212] Kim S. P., Choi M. Y., Choi H. C. (2015). Appl. Surf. Sci..

[cit213] Lu Z., Wang H. (2014). CrystEngComm.

[cit214] Song H., Li N., Cui H., Wang C. (2014). Electrochim. Acta.

[cit215] Deng J., Dai Y., Xiao Z., Song S., Dai H., Li L., Li J. (2020). Nanomaterials.

[cit216] Tian Q. H., Chen Y. B., Zhang W., Sui Z. Y., Yang L. (2020). J. Alloys Compd..

[cit217] Zhou X., Yu L., Lou X. W. (2016). Nanoscale.

[cit218] Yang A., Tao X., Wang R., Lee S., Surya C. (2007). Appl. Phys. Lett..

[cit219] Li X.-H., Huang H.-C., Ling L., Wang X.-Y., Zhang J.-R., Gao L. (2011). Chin. J. Inorg. Chem..

[cit220] Xia M., Guo H.-Y., Yang B. (2018). Fullerenes, Nanotubes, Carbon Nanostruct..

[cit221] Wu P., Du N., Zhang H., Yu J., Qi Y., Yang D. (2011). Nanoscale.

[cit222] Du G., Zhong C., Zhang P., Guo Z., Chen Z., Liu H. (2010). Electrochim. Acta.

[cit223] Liu K., Zhu S., Dong X., Huang H., Qi M. (2020). Adv. Mater. Interfaces.

[cit224] Jin Y.-H., Min K.-M., Seo S.-D., Shim H.-W., Kim D.-W. (2011). J. Phys. Chem. C.

[cit225] Liang G., Sun X., Lai J., Wei C., Huang Y., Hu H. (2019). J. Electroanal. Chem..

[cit226] Tian Q., Tian Y., Zhang Z., Yang L., Hirano S.-i. (2015). J. Power Sources.

[cit227] Liu M., Zhang S., Dong H. C., Chen X., Gao S., Sun Y. P., Li W. H., Xu J. Q., Chen L. W., Yuan A. B., Lu W. (2019). ACS Sustainable Chem. Eng..

[cit228] Liu Q., Dou Y., Ruan B., Sun Z., Chou S. L., Dou S. X. (2016). Chemistry.

[cit229] Wan Y., Sha Y., Deng W., Zhu Q., Chen Z., Wang X., Chen W., Xue G., Zhou D. (2015). Electrochim. Acta.

[cit230] He F. R., Xu Q., Zheng B. P., Zhang J., Wu Z. G., Zhong Y. J., Chen Y. X., Xiang W., Zhong B. H., Guo X. D. (2020). RSC Adv..

[cit231] Yuan L., Konstantinov K., Wang G. X., Liu H. K., Dou S. X. (2005). J. Power Sources.

[cit232] Liu H., Long D., Liu X., Qiao W., Zhan L., Ling L. (2009). Electrochim. Acta.

[cit233] Shi J., Lin N., Liu D., Wang Y., Lin H. (2020). J. Electroanal. Chem..

[cit234] Sun X., Liu J., Li Y. (2006). Chem. Mater..

[cit235] Qiao H., Zheng Z., Zhang L., Xiao L. (2008). J. Mater. Sci..

[cit236] Tao X., Tian Q., Yang L., Xiang Y. (2017). Mater. Lett..

[cit237] Wu P., Du N., Zhang H., Zhai C., Yang D. (2011). ACS Appl. Mater. Interfaces.

[cit238] Chen Y., Huang Q. Z., Wang J., Wang Q., Xue J. M. (2011). J. Mater. Chem..

[cit239] Wang M.-S., Lei M., Wang Z.-Q., Zhao X., Xu J., Yang W., Huang Y., Li X. (2016). J. Power Sources.

[cit240] Wu Z.-G., Li J.-T., Zhong Y.-J., Liu J., Guo X.-D., Huang L., Zhong B.-H., Sun S.-G. (2015). J. Alloys Compd..

[cit241] Wang X., Fan H., Ren P., Li M. (2014). RSC Adv..

[cit242] Zhao X., Wen T., Zhang J., Ye J., Ma Z., Yuan H., Ye X., Wang Y. (2017). RSC Adv..

[cit243] Yuan J., Chen C., Hao Y., Zhang X., Zou B., Agrawal R., Wang C., Yu H., Zhu X., Yu Y., Xiong Z., Luo Y., Li H., Xie Y. (2017). J. Alloys Compd..

[cit244] Ma C., Zhang W., He Y. S., Gong Q., Che H., Ma Z. F. (2016). Nanoscale.

[cit245] Yang S.-L., Zhou B.-H., Lei M., Huang L.-P., Pan J., Wu W., Zhang H.-B. (2015). Chin. Chem. Lett..

[cit246] Yiliguma, Wang Z., Yang C., Guan A., Shang L., Al-Enizi A. M., Zhang L., Zheng G. (2018). J. Mater. Chem. A.

[cit247] Nam S., Kim S., Wi S., Choi H., Byun S., Choi S.-M., Yoo S.-I., Lee K. T., Park B. (2012). J. Power Sources.

[cit248] Ji X., Huang X., Liu J., Jiang J., Li X., Ding R., Hu Y., Wu F., Li Q. (2010). Nanoscale Res. Lett..

[cit249] Yu L., Cai D., Wang H., Titirici M.-M. (2013). RSC Adv..

[cit250] Lou X. W., Chen J. S., Chen P., Archer L. A. (2009). Chem. Mater..

[cit251] Courtel F. M., Baranova E. A., Abu-Lebdeh Y., Davidson I. J. (2010). J. Power Sources.

[cit252] Xu G.-L., Chen S.-R., Li J.-T., Ke F.-S., Huang L., Sun S.-G. (2011). J. Electroanal. Chem..

[cit253] Zhang W., Liu H. (2017). Neurocomputing.

[cit254] Hernández-Rentero C., Marangon V., Olivares-Marín M., Gómez-Serrano V., Caballero Á., Morales J., Hassoun J. (2020). J. Colloid Interface Sci..

[cit255] Dou Y., Liu X., Yu K., Wang X., Liu W., Liang J., Liang C. (2019). Diamond Relat. Mater..

[cit256] Hu L.-L., Yang L.-P., Zhang D., Tao X.-S., Zeng C., Cao A.-M., Wan L.-J. (2017). Chem. Commun..

[cit257] Tian Q., Zhang F., Zhang W., Yang L. (2019). Electrochim. Acta.

[cit258] Rujia Z., Zhang Z., Jiang L., Xu K., Tian Q., Xue S., Hu J., Bando Y., Golberg D. (2012). J. Mater. Chem..

[cit259] Zhang W., Li M., Xiao X., Huang X., Jiang Y., Fan X., Chen L. (2017). J. Alloys Compd..

[cit260] Wu Z. S., Sun Y., Tan Y. Z., Yang S., Feng X., Mullen K. (2012). J. Am. Chem. Soc..

[cit261] Stankovich S., Dikin D. A., Piner R. D., Kohlhaas K. A., Kleinhammes A., Jia Y., Wu Y., Nguyen S. T., Ruoff R. S. (2007). Carbon.

[cit262] Castro Neto A. H., Guinea F., Peres N. M. R., Novoselov K. S., Geim A. K. (2009). Rev. Mod. Phys..

[cit263] Geim A. K. (2009). Science.

[cit264] Zuo S.-y., Wu Z.-g., Li S.-k., Yan D., Liu Y.-h., Wang F.-y., Zhuo R.-f., Geng B.-s., Wang J., Yan P.-x. (2017). RSC Adv..

[cit265] Zhou D., Li X., Fan L.-Z., Deng Y. (2017). Electrochim. Acta.

[cit266] Zhang M., Sun Z., Zhang T., Sui D., Ma Y., Chen Y. (2016). Carbon.

[cit267] Li Z., Wu G., Deng S., Wang S., Wang Y., Zhou J., Liu S., Wu W., Wu M. (2016). Chem. Eng. J..

[cit268] Han J., Kong D., Lv W., Tang D. M., Han D., Zhang C., Liu D., Xiao Z., Zhang X., Xiao J., He X., Hsia F. C., Zhang C., Tao Y., Golberg D., Kang F., Zhi L., Yang Q. H. (2018). Nat. Commun..

[cit269] Shi S., Deng T., Zhang M., Yang G. (2017). Electrochim. Acta.

[cit270] Wu Z.-S., Ren W., Xu L., Li F., Cheng H.-M. (2011). ACS Nano.

[cit271] Liu D. D., Wei Z. Y., Zhong B., Liu L. M., Zhang T., Duan X. M., Chen M., Wang H. T., Huang X. X. (2020). Appl. Surf. Sci..

[cit272] Huang Y., Yu R., Mao G., Yu W., Ding Z., Cao Y., Zheng J., Chu D., Tong H. (2020). J. Alloys Compd..

[cit273] Zhang H., Liu S. (2020). J. Alloys Compd..

[cit274] Wang K., Li Z. (2020). J. Nanosci. Nanotechnol..

[cit275] Liu D., Wei Z., Zhong B., Liu L., Zhang T., Duan X., Chen M., Wang H., Huang X. (2020). Appl. Surf. Sci..

[cit276] Zuo X., Li B., Chang K., Tang H., Chang Z. (2017). RSC Adv..

[cit277] Usachov D., Vilkov O., Gruneis A., Haberer D., Fedorov A., Adamchuk V. K., Preobrajenski A. B., Dudin P., Barinov A., Oehzelt M., Laubschat C., Vyalikh D. V. (2011). Nano Lett..

[cit278] Song H., Li N., Cui H., Wang C. (2014). Nano Energy.

[cit279] Zhou X., Wan L. J., Guo Y. G. (2013). Adv. Mater..

[cit280] Wu N., Du W., Gao X., Zhao L., Liu G., Liu X., Wu H., He Y. B. (2018). Nanoscale.

[cit281] Paraknowitsch J. P., Thomas A. (2013). Energy Environ. Sci..

[cit282] Wu K., Shi B., Qi L., Mi Y., Zhao B., Yang C., Wang Q., Tang H., Lu J., Liu W., Zhou H. (2018). Electrochim. Acta.

[cit283] Maiti U. N., Lee W. J., Lee J. M., Oh Y., Kim J. Y., Kim J. E., Shim J., Han T. H., Kim S. O. (2014). Adv. Mater..

[cit284] Verchere A., Mishra S., Jeanneau E., Guillon H., Decams J.-M., Daniele S. (2020). Inorg. Chem..

[cit285] Sun Y., Cheng S., Yu Z., Li L., Li C., Yang J. (2020). J. Alloys Compd..

[cit286] Maleki M. (2020). Acta Phys. Pol., A.

[cit287] Wang W., Liu Y., Liu S. (2020). Cryst. Growth Des..

[cit288] Ma C. R., Zhang W. M., He Y. S., Gong Q., Che H. Y., Ma Z. F. (2016). Nanoscale.

[cit289] Wen L., Li F., Cheng H.-M. (2016). Adv. Mater..

[cit290] Zhang B. A., Zheng Q. B., Huang Z. D., Oh S. W., Kim J. K. (2011). Carbon.

[cit291] Wang J., Fang F., Yuan T., Yang J., Chen L., Yao C., Zheng S., Sun D. (2017). ACS Appl. Mater. Interfaces.

[cit292] Kulshreshtha S. K., Gadgil M. M., Sasikala R. (1996). Catal. Lett..

[cit293] Jayalakshmi A., Venugopal N., Raja K. P., Rao M. M. (2006). J. Power Sources.

[cit294] Shin S.-H., Song J.-M., Kim S.-W., Shin J.-K., Lee S.-M., Ro J.-C., Suh S.-J. (2020). Phys. Status Solidi A.

[cit295] Shao D., Zhang X., Wang Z., Zhang Y., Tan G., Yan W. (2020). Appl. Surf. Sci..

[cit296] Shao D., Zhang Y., Lyu W., Zhang X., Tan G., Xu H., Yan W. (2020). J. Hazard. Mater..

[cit297] Liu M., Fan H., Zhuo O., Chen J., Wu Q., Yang L., Peng L., Wang X., Che R., Hu Z. (2020). Nano Energy.

[cit298] Luo G., Liu W., Zeng S., Zhang C., Yu X., Fang Y., Sun L. (2015). Electrochim. Acta.

[cit299] Liu Y., Jiao Y., Yin B., Zhang S., Qu F., Wu X. (2015). J. Mater. Chem. A.

[cit300] Tian Q., Yan J., Yang L., Chen J. (2018). Electrochim. Acta.

[cit301] Xie H., Chen M., Wu L. (2017). Small.

[cit302] Yang H., Song T., Lee S., Han H., Xia F., Devadoss A., Sigmund W., Paik U. (2013). Electrochim. Acta.

[cit303] Chen J. S., Li C. M., Zhou W. W., Yan Q. Y., Archer L. A., Lou X. W. (2009). Nanoscale.

[cit304] Wang Y., Xu J., Wu H., Xu M., Peng Z., Zheng G. (2012). J. Mater. Chem..

[cit305] Zhang D.-F., Sun L.-D., Jia C.-J., Yan Z.-G., You L.-P., Yan C.-H. (2005). J. Am. Chem. Soc..

[cit306] Wang W.-W. (2008). Mater. Res. Bull..

[cit307] Zhou W., Cheng C., Liu J., Tay Y. Y., Jiang J., Jia X., Zhang J., Gong H., Hng H. H., Yu T., Fan H. J. (2011). Adv. Funct. Mater..

[cit308] Chen Z. X., Zhou M., Cao Y. L., Ai X. P., Yang H. X., Liu J. (2012). Adv. Energy Mater..

[cit309] Yuan T., Jiang Y., Li Y., Zhang D., Yan M. (2014). Electrochim. Acta.

[cit310] Wu W., Zhao Y., Li J., Wu C., Guan L. (2014). J. Energy Chem..

[cit311] Guo J., Zhu H., Sun Y., Tang L., Zhang X. (2016). J. Mater. Chem. A.

[cit312] Park G. D., Lee J. K., Kang Y. C. (2017). J. Mater. Chem. A.

[cit313] Koo B.-R., Lee D.-H., Ahn H.-J., Sung Y.-E. (2016). J. Nanosci. Nanotechnol..

[cit314] Ma J., Li J., Yang S., Lu H., Liu L., Wang R. (2020). J. Power Sources.

[cit315] Qi Y., Zhang H., Du N., Zhai C., Yang D. (2012). RSC Adv..

[cit316] Hou J., Cao C., Idrees F., Ma X. (2015). ACS Nano.

[cit317] Sun Y. N., Goktas M., Zhao L., Adelhelm P., Han B. H. (2020). J. Colloid Interface Sci..

[cit318] Li X., Zhang Y., Li T., Zhong Q., Li H., Huang J. (2014). Electrochim. Acta.

[cit319] Tao W., Wang M., Zhu B., Huo W., Yang R., Xiong H., Tang H., Wei Z., Wang Y. (2020). Electrochim. Acta.

[cit320] Huang B., Pan Z., Su X., An L. (2018). J. Power Sources.

[cit321] Zhou H., Zhong Y., He Z., zhang L., Wang J., Zhang J., Cao C.-n. (2014). Appl. Surf. Sci..

[cit322] Ding J., Gao X., Cha L., Cai M.-Q., Ma J. (2016). RSC Adv..

[cit323] Tian Q., Zhang Z., Yang L., Hirano S.-i. (2015). RSC Adv..

[cit324] Yang F., Zhu Y., Li X., Lai C., Guo W., Ma J. (2015). RSC Adv..

[cit325] Xing Y., Wang S., Fang B., Song G., Wilkinson D. P., Zhang S. (2018). J. Power Sources.

[cit326] Shannon R. D. (1976). Acta Crystallogr., Sect. A: Cryst. Phys., Diffr., Theor. Gen. Crystallogr..

[cit327] Zhou L., Guo H., Li T., Chen W., Liu L., Qiao J., Zhang J. (2015). Sci. Rep..

[cit328] Yang Z., Du G., Guo Z., Yu X., Chen Z., Guo T., Zeng R. (2011). Nanoscale.

[cit329] Yi Z., Han Q., Zan P., Cheng Y., Wu Y., Wang L. (2016). J. Mater. Chem. A.

[cit330] Cheong J. Y., Kim C., Jang J. S., Kim I.-D. (2016). RSC Adv..

[cit331] Xu A., Dai X., Wei K., Han W., Li J., Sun X., Shen J., Wang L. (2017). RSC Adv..

[cit332] Xu L., Wang Y., Zhang W. (2019). RSC Adv..

[cit333] Yang Z., Du G., Meng Q., Guo Z., Yu X., Chen Z., Guo T., Zeng R. (2011). RSC Adv..

[cit334] Yoo H., Lee G., Choi J. (2019). RSC Adv..

[cit335] Yang Z., Meng Q., Guo Z., Yu X., Guo T., Zeng R. (2013). J. Mater. Chem. A.

[cit336] He L., Wang C., Yao X., Ma R., Wang H., Chen P., Zhang K. (2014). Carbon.

[cit337] Lu Z., Wang H. (2014). CrystEngComm.

[cit338] Tang Y., Wu D., Chen S., Zhang F., Jia J., Feng X. (2013). Energy Environ. Sci..

[cit339] Bai S., Shen X. (2012). RSC Adv..

[cit340] Singh R. K., Kumar R., Singh D. P. (2016). RSC Adv..

[cit341] Xiang H. F., Li Z. D., Xie K., Jiang J. Z., Chen J. J., Lian P. C., Wu J. S., Yu Y., Wang H. H. (2012). RSC Adv..

[cit342] Zhao G., Wen T., Chen C., Wang X. (2012). RSC Adv..

[cit343] Abbasnezhad A., Asgharzadeh H., Ansari Hamedani A., Hayat Soytas S. (2020). Dalton Trans..

[cit344] Bai S., Chen S., Shen X., Zhu G., Wang G. (2012). RSC Adv..

[cit345] Tao L., Zai J., Wang K., Wan Y., Zhang H., Yu C., Xiao Y., Qian X. (2012). RSC Adv..

[cit346] Du C. Y., Chen M., Cao X. Y., Yin G. P., Shi P. F. (2009). Electrochem. Commun..

[cit347] Zhao J., Shan W., Xia X., Wang Q., Xing L. (2014). Sci. China: Technol. Sci..

[cit348] Sun J., Wang Q., Wang Q., Zhang D. A., Xing L. L., Xue X. Y. (2016). Sci. Adv. Mater..

[cit349] Cai R., Yu X., Liu X., Shao Z. (2010). J. Power Sources.

[cit350] Hui Y., Cao L., Xu Z., Huang J., Ouyang H., Li J. (2016). J. Electroanal. Chem..

[cit351] Gao J., Jiang C., Ying J., Wan C. (2006). J. Power Sources.

[cit352] Song K., Seo D.-H., Jo M. R., Kim Y.-I., Kang K., Kang Y.-M. (2014). J. Phys. Chem. Lett..

[cit353] Mueller F., Bresser D., Chakravadhanula V. S. K., Passerini S. (2015). J. Power Sources.

[cit354] Chen A., Xia S. J., Pan H. Y., Xi J. H., Qin H. Y., Lu H. W., Ji Z. G. (2018). J. Electroanal. Chem..

[cit355] Cui Y. H., Feng Y. J. (2005). J. Mater. Sci..

[cit356] Subramanian V., Gnanasekar K. I., Rambabu B. (2004). Solid State Ionics.

[cit357] Jia T., Chen J., Deng Z., Fu F., Zhao J., Wang X., Long F. (2014). Mater. Sci. Eng., B.

